# Voxel Volume Overlap: Voxel‐Size Sensitive Indicators of Subject Motion in Functional MRI

**DOI:** 10.1002/hbm.70337

**Published:** 2025-09-09

**Authors:** Marko Wilke

**Affiliations:** ^1^ Department of Neuropediatrics, General Pediatrics, Diabetology, Endocrinology, Social Pediatrics University Children's Hospital Tübingen Germany; ^2^ Experimental Pediatric Neuroimaging, Children's Hospital and Department of Neuroradiology University of Tübingen Tübingen Germany

**Keywords:** functional MRI, multisite studies, out‐of‐plane motion, subject motion, volume overlap

## Abstract

Subject motion is a significant problem for the analysis of functional MRI data and is usually described by “total displacement” or “scan‐to‐scan displacement”. Neither parameter, however, takes into account voxel size, which clearly is relevant for the actual effects of motion on the data. Consequently, it is hitherto impossible to compare motion between subjects/studies acquired using different voxel dimensions, precluding the development of generally applicable recommendations for fMRI quality control procedures. Here, a new set of “voxel volume overlap” (VVO) parameters is developed and explored, assessing the actual volumetric effects of subject motion on the voxel‐level. Further, the extent of out‐of‐plane movement (particularly detrimental to image quality) can be quantified. Analyses show the new parameters to be valid and sensitive to voxel sizes. Their relation to existing parameters is explored, and defaults are suggested. The algorithm is freely available as a toolbox for a common image processing software solution (SPM).

## Graphical abstract

Different impact of identical motion in fMRI solely due to voxel sizes, as detected by scan‐to‐scan voxel volume overlap (STS_VVO_; > 90% overlap: green, > 80%: yellow, > 55%: red, < 55%: gray), for 3 × 3 × 3 (A & B) vs. 2 × 2 × 2 mm (C & D). Note substantial shifts toward lower overlap with higher spatial resolution.

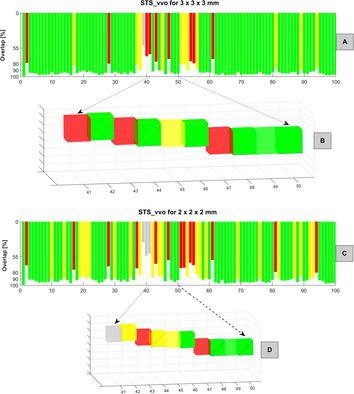

## 1 Introduction

Vulnerability to subject motion is a significant issue when performing functional MRI in humans (Bause et al. 2020; Lemieux et al. 2007; Lund et al. 2005; Murphy et al. 2013; Power et al. 2012). Numerous contributing sources have been identified, including physiological, technical, and subject factors (Godenschweger et al. 2016; Liu 2016; Murphy et al. 2013; Zaitsev et al. 2015). Since the very early days of fMRI, a multitude of approaches were developed to “correct” for subject motion: post hoc (still the most commonly‐used approach; Friston et al. 1996; Jenkinson et al. 2002), on the raw data level (Loktyushin et al. 2015), or by accounting for it in statistical analyses (Lemieux et al. 2007; Satterthwaite et al. 2013; Wilke and Baldeweg 2019). However, all are known to only incompletely remove motion effects (Andersson et al. 2001; Wu et al. 1997).

Along the way, our understanding of how subject motion should be described has increased, moving away from simply looking at translation (Wilke 2014) to more comprehensive indicators of total displacement (TD) and, more importantly, scan‐to‐scan displacement (STS; Power et al. 2012). While several approaches to describe “data quality” in a broader sense were developed (Afyouni and Nichols 2018; Smyser et al. 2010; Power 2017), absolute thresholds of subject motion are still commonly used to identify and remove motion‐corrupted datapoints (Morfini et al. 2023; Power et al. 2014; Satterthwaite et al. 2013; Smyser et al. 2010; Williams and Lindner 2020). This approach is in widespread use, although it is acknowledged as being heuristic, not empirical (Parkes et al. 2018).

One factor that has been curiously neglected when describing and/or judging subject motion is the voxel size used when acquiring fMRI data. Traditionally, motion exceeding one (Mattern et al. 2018; Wilke et al. 2005; Williams and Lindner 2020) or half of one voxel size (Power et al. 2012) was considered unacceptable, and the relevance of voxel size was discussed in the context of partial volume effects (e.g., Zaitsev et al. 2017) and signal‐to‐noise considerations (e.g., Murphy et al. 2007; Newton et al. 2012). Otherwise, this factor does not seem to have been explored in greater depths in this particular context. This is somewhat surprising as it is immediately apparent that the same amount of subject motion must have dramatically different effects on the level of the smallest spatial component, the individual voxel, depending on this voxel's dimensions. For example, a simple shift of 1 mm in one dimension from one timepoint to the next will lead to the second timepoint overlapping with the first timepoint in 18/27 mm^3^ in the case of a 3 × 3 × 3 mm voxel, corresponding to 66.6%. In other words, of the volume covered by the first voxel, two‐thirds also contribute to the second timepoint. In the case of a 2 × 2 × 2 mm voxel, that value is 4/8 mm^3^, that is, 50%, while it is 0% in the case of a 1 × 1 × 1 mm voxel. Effectively, therefore, the tissue imaged at timepoint 2 will either be “largely overlapping” or “completely different”, depending not on the amount of subject motion, but on the spatial resolution of the data. Quite obviously and as noted before, “any alteration in voxel content will manifest itself as a change in the BOLD signal” (Murphy et al. 2013), and the impact of such “partial‐volume effect modulation” was considered “high” (Zaitsev et al. 2017). While it can convincingly be argued that the actual (effective) smoothness of fMRI data is different (see below), the effect itself remains. Therefore, motion parameters (translations and rotations as well as TD and STS) are not comparable between subjects/studies, and the strictness of quality control approaches cannot be compared either, if voxel sizes are not identical. This precludes generalizable recommendations across studies. The aim of the current manuscript therefore was to develop and explore new indicators for subject motion that take voxel size into account.

## 2 Subjects and Methods

*Dataset 1* As an example of high quality resting state data, imaging data from the fCONN1000 study was used (http://fcon_1000.projects.nitrc.org/). In total, 712 imaging sessions were used, one each per subject, with an average of 182 ± 73 (range, 115–395) scans per session, resulting in 129.850 datapoints. Mean voxel size was 3.18 ± 0.42 (range, 2–4) mm in X, 3.18 ± 0.42 (range, 2–4) mm in Y, and 3.55 ± 0.62 (range, 3–5.5) mm in Z, and mean scan‐to‐scan subject motion was 0.0966 ± 0.0808 mm (range, 0.0258–1.1915). This dataset was acquired in a total of 11 locations on healthy adults and has the advantage of lacking task‐related activation and task‐related motion (Biswal and Hyde 1997; Johnstone et al. 2006; Lund et al. 2005).

*Dataset 2* As an example of high‐quality task‐based data, the second dataset is from a previous study conducted on adults in Tübingen to optimize an fMRI task battery (Máté et al. 2016). In total, 80 imaging sessions were available, four each per subject, with a final 100 scans per session, resulting in 8.000 datapoints. Voxel size was 3 × 3 × 3 mm and mean scan‐to‐scan subject motion was 0.0742 ± 0.0602 mm (range, 0.0251–0.437). It was acquired in one location (University hospital Tübingen) from healthy adults and consisted of three language tasks and one active motion task (bilateral foot movement vs. rest). It was approved by the ethics committee of the University Hospital Tübingen (518/2013/BO2).

*Dataset 3* As an example of a low‐quality task‐based data, the third dataset is from a previous study conducted in Tübingen on children in the presurgical context. In total, 100 imaging sessions were available, with 100 scans per session, resulting in 10,000 datapoints. Voxel size was 3 × 3 × 3 mm and mean scan‐to‐scan subject motion was 0.2625 ± 0.6803 mm (range, 0.0316.3975). It was acquired in one location (University hospital Tuebingen) from children being assessed for potential neurosurgical intervention and consisted of several different language tasks (Wilke et al. 2011, 2018). It was approved by the ethics committee of the University Hospital Tübingen (420/2012/BO1). More details on each dataset can be found in the Supporting Information 4.

All processing and analysis steps were done employing functionality available within SPM12 (Wellcome Department of Imaging Neuroscience, University College London, UK) or using custom scripts and functions, running within Matlab (The Mathworks, Natick, USA). Minimizing interpolation artifacts was achieved by using 7th degree B‐spline interpolation (Unser 1999) wherever possible. Realignment was carried out to correct for subject motion (Friston et al. 1996), with the “quality flag” in SPM12 set to maximum. No temporal interpolation (such as slice timing) was performed as this may interfere with motion detection (Power et al. 2017).

*Calculation of total displacement and scan‐to‐scan displacement*: Motion is usually “corrected for” by applying a rigid‐body translation to all timepoints, minimizing the difference with regard to the first (or mean) volume in a timeseries (Friston et al. 1996; Jenkinson et al. 2002). While the exact implementation differs slightly between software solutions, the approaches are very comparable (Hoffmann et al. 2015). They result in a set of six parameters describing said motion, namely translation in each direction (termed X, Y, and Z) and rotations around each axis (termed “pitch”, “roll”, and “yaw”). Of note, while shifts are identically applied to the whole volume, rotations induce actual translation as a function of distance from the origin around which the volume is rotated (the further away, the larger translation will be). Hence, it is necessary to define an exemplary distance; to this effect, the empirically derived value of 65 mm was used (Wilke 2012), reflecting the average cortical distance in adults (Wilke 2014).

When calculating subject motion, it is important to be precise about the respective reference frame. If motion is calculated w.r.t. the first image in a timeseries, this measure is known as total displacement (TD). It describes how far away from the original position a subject moved in the course of the session. This, however, is not necessarily the most relevant measure, as slow movements are more easily accounted for than rapid motion spikes (Maknojia et al. 2019). For the latter in particular, scan‐to‐scan displacement (STS) is more relevant, which uses the individually preceding volume as the reference frame. The way these two indicators are calculated is similar, using a 3D expansion of Pythagoras theorem (Wilke 2014) to calculate the joint displacement resulting from all shifts and all rotations.

### 2.1 Open Questions

While it is trivial to estimate the volumetric effects of simple shifts (see example in the introduction), it is far from trivial to account for rotations as there is a multitude of possible rotatory effects, and their combinations, on a 3D volume. Computing the exact overlap between two arbitrarily‐rotated cubic volumes, therefore, is mathematically highly challenging. However, while no elegant algebraic solution exists, it is possible to assess the overlap by using a geometric approach. Here, the reference voxel is defined as the intersection of six planar boundaries, with each plane dividing space into two categories: “definitely outside” and “potentially inside”; the intersection of all “potentially inside” volumes then unequivocally defines the cube. For example, consider a dice on a table and its top surface showing, for example, “1”. Everything above that surface is outside of the dice, while everything below *may* be inside. The lower (opposite) surface will then be defined by the face of the dice showing “6”, which in our example is facing the table. Again, everything below that surface is outside. Repeating this process of exclusion with the “4 & 3” and the “2 & 5” faces will unequivocally define the space, and hence, the volume of the dice. The intersection of a moved cube with a reference cube can therefore be calculated by iteratively looping over all six planes (of the reference cube) and by therewith removing all “definitely outside” sections (of the moved cube). The remaining part constitutes the overlapping remnant of the moved cube, the volume of which can then be computed.

*Algorithmic implementation*: Comparing the moved cube to the reference cube was achieved as follows: each reference cube is described by six planes, constituting inequality constraints. Each plane is defined by one point on each surface and a corresponding norm vector, defining “inside” (and consecutively, of course, “outside”), establishing a set of six cutting planes. The reference cube is then moved, using the rigid‐body parameters from the realignment procedure. This creates the moved cube described by its eight (moved) corners and their connections, which again are vectors. The intersection of these vectors with a given cutting plane can be calculated using linear interpolation, resulting in a new set of (cut) vectors, jointly defining a new (cut) surface. The iterative application of all six cutting planes will remove all parts of the moved cube that are “outside” of the reference voxel's boundaries. The coordinates of the remaining shape can then be used to generate a convex 3D shape using Delauney triangulation. As rotations are involved, the shape may be irregular, but it is a polygon, and every polygon can be triangulated (de Berg et al. 2008). This triangulation will yield a set of tetrahedra whose (sum of) volumes can be calculated. The volume of this remnant of the moved cube can then finally be related to the volume of the reference cube.

### 2.2 Resulting Measures

As mentioned above, the reference for conventional motion measures is either the first volume, resulting in total displacement (TD), or the immediately preceding volume, resulting in scan‐to‐scan (STS) motion. Similarly, two overlap measures are calculated for each voxel: using the first voxel or the preceding voxel as reference results in a “total displacement voxel volume overlap” (TD_VVO_) or a “scan‐to‐scan voxel volume overlap” (STS_VVO_) measure, respectively. Both measures are expressed in percent, indicating the percentage of the moved voxel's volume overlap with the first (TD_VVO_) or the preceding volume (STS_VVO_), respectively.

Further, as the of non‐overlapping parts of the moved cube can also be determined, assessing these on opposite sides of the reference volume allows one to assess the exact proportion of in‐plane versus out‐of‐plane motion (Maknojia et al. 2019; Power et al. 2017; Zaitsev et al. 2017). For example, the volume of the moved cube found superior and inferior to the original voxel location (in the case of an axial acquisition) is out‐of‐plane, while all other movements (in left–right, or anterior–posterior directions) result in in‐plane volume changes. These values can also be related to each other, expressing (in %) the proportion of out‐of‐plane motion.

### 2.3 Practicality Considerations

As motion effects are not uniform across the image volume (Power 2017; Wilke 2012, 2014), these steps would have to be repeated for each voxel and each timepoint to fully describe motion effects. While it is possible, it is not practical to do so. Hence, a set of six representative reference voxels located at the average cortical distance (65 mm; Wilke 2014) was used: anterior and posterior, right and left, and superior and inferior to the reference volume's origin.

In Experiment 1, several basic and unique features of the newly proposed measures were explored. In Experiment 2, their relation with existing measures was investigated. For each experiment, all datasets were used and results were displayed individually as well as jointly (when meaningful) to explore possible effects of data quality.

### 2.4 Experiment 1a

To assess the validity and the accuracy of the approach, a set of simulations was run. To establish a ground truth, “motion parameters” were generated consisting of simple shifts, with a magnitude of 0.5, 1, 1.5, 2, 2.5, and 3 mm, in each dimension. The effect of these “movements” was then assessed on simulated voxel sizes of 4 × 4 × 4 mm, 3.5 × 3.5 × 3.5 mm, 3 × 3 × 3 mm, 2.5 × 2.5 × 2.5 mm, 2 × 2 × 2 mm, 1.5 × 1.5 × 1.5 mm, 1 × 1 × 1 mm, and 0.5 × 0.5 × 0.5 mm. Expected and observed volumetric overlap were then calculated and compared.

### 2.5 Experiment 1b

To assess how far the six representative voxels show different effects of motion, both overlap measures were derived for each of the six locations in each subject and were then correlated with the individual mean values (over all 6 locations).

### 2.6 Experiment 1c

To assess the voxel‐size sensitivity of TD_VVO_ and STS_VVO_, simulations were run using the actual motion parameters, but assuming different voxel sizes of 4 × 4 × 4 mm, 3.5 × 3.5 × 3.5 mm, 3 × 3 × 3 mm, 2.5 × 2.5 × 2.5 mm, 2 × 2 × 2 mm, 1.5 × 1.5 × 1.5 mm, 1 × 1 × 1 mm, and 0.5 × 0.5 × 0.5 mm.

### 2.7 Experiment 1d

While the acquired resolution of the data is determined by the voxel size, the effective spatial resolution in fMRI data is determined by the smoothness of the data. In ensuing statistical analyses, this also determines the number of resolution elements (“resels”) used to account for multiple comparisons (Kiebel et al. 1999). Of note, while this smoothness of course is nonstationary across the image volume (Hayasaka et al. 2004), an average indicator is commonly used to approximate the mean effective resolution of the data, which can be derived for each dimension. Within the SPM12 software package, this is calculated from the standardized residuals of the general linear model (Kiebel et al. 1999; Worsley 1996), but similar tools exist for other software solutions such as AFNI or FSL, taking only slightly different approaches (Eklund et al. 2016). The effective resolution was compared to the acquired resolution of the data in each spatial dimension, and the effect of using the effective resolution of the data on the overlap measures was explored.

### 2.8 Experiment 1e

As noted above, movement within the acquisition plane will have different effects than out‐of‐plane movement (Power et al. 2017), particularly due to spin‐history effects (Maknojia et al. 2019; Zaitsev et al. 2017). Hence, describing the magnitude of these out‐of‐plane shifts in more detail would be interesting. To this effect, instead of assessing the volume overlap, the proportion of the volume *not* overlapping with the reference voxel, outside of the original acquisition plane, was calculated instead. For an axial acquisition, as here, this corresponds to values above and below the original voxel; for coronal acquisition, before and behind, and for sagittal, left and right of it. To assess the effect of the acquisition plane on out‐of‐plane shifts, all datasets were analyzed “as if” acquired in an axial, coronal, or sagittal acquisition, and the resulting proportion of out‐of‐plane volume shifts was recorded. Of note, as the acquisition plane does not change over time, this parameter was only calculated using the first volume as its reference.

### 2.9 Experiment 2a

STS_VVO_ was related to the percent change in the magnitude of the fast variance component of DVARS (Δ%D‐VAR; Afyouni and Nichols 2018) which was obtained using a previously published algorithm (Wilke and Baldeweg 2019). The correlation between Δ%D‐VAR and STS_VVO_ was calculated for each timepoint and each subject, as, again, both parameters use the preceding timepoint as the reference, making them comparable.

### 2.10 Experiment 2b

TD_VVO_ and STS_VVO_ from each timepoint and each subject were related to the corresponding conventional measures (TD and STS; Power et al. 2012; Wilke 2014). Additionally, their correlations were calculated for each subject.

### 2.11 Experiment 2c

Following up on Experiment 1c as well as Experiment 2b, the correspondence of the absolute values of STS and STS_VVO_ was assessed for all simulated voxel sizes as defined for Experiment 1c and in all datasets.

### 2.12 Experiment 2d

The concrete mathematical relation between STS and STS_VVO_ was assessed in a subset of 392 sessions, all of which were acquired with the same spatial resolution (3 × 3 × 3 mm; 218 from dataset 1, 80 from dataset 2, and 94 from dataset 3). For each of these sessions, a linear fit between clinically realistic STS thresholds from 0 to 1.5 mm (Wilke and Baldeweg 2019) with the corresponding STS_VVO_ values was performed, using:

y=ax+b

The fit was mathematically constrained (Mjaavatten 2024) such that a movement of 0 had to result in an overlap of 100%. The coefficient *a* describing this linear relation was obtained if more than 25% of the original datapoints survived, and the mean coefficient over all sessions was used to mathematically derive STS_VVO_ values corresponding to the given STS values (again in the range from 0 to 1.5 mm).

### 2.13 Statistical Analyses

For all analyses, significance was assumed at *p* ≤ 0.05, Bonferroni‐corrected for multiple comparisons where appropriate. The assumption of normality was initially tested using a Kolmogorov–Smirnov‐Liliefors‐Test. If this assumption was met, a two‐sided Student's *T*‐Test was run, and data was described using mean and standard deviation. Otherwise, a Mann‐Whitney‐Test was run, and results were expressed as medians and median absolute deviation (MAD). For correlation analyses, a Spearman correlation coefficient was used for normally distributed data, and a Kendall rank correlation otherwise. All calculations were performed in MATLAB (The Mathworks).

## 3 Results

### 3.1 Experiment 1a

Assessing the validity and accuracy of the approach using simulated shifts, the expected and observed volumetric changes are listed in Table 1. For all shifts and all voxel locations, observed results were in full agreement with the expected values, up to the fourth decimal place.

**TABLE 1:** Illustration of expected (E) percent volume overlap and difference between expected and observed values (ΔEO), for 6 simulated shifts (columns) and 8 simulated isotropic voxel sizes (rows). For example, a shift of 2 mm (column 9) of an isotropic voxel of 4 mm (row 4) will result in a voxel volume overlap of 50% (in bold). Note substantial impact of voxel size on resulting overlap, and full correspondence of expected and observed values in all cases. See text for details.

		Simulated shifts [mm]											
		0.5		1		1.5		2		2.5		3	
		E	ΔEO	E	ΔEO	E	ΔEO	E	ΔEO	E	ΔEO	E	ΔEO
**Simulated voxel size [mm]**	**4**	87.5	0	75	0	62.5	0	**50**	0	37.5	0	25	0
	**3.5**	85.7142	0	71.4285	0	57.1428	0	42.8571	0	28.5714	0	14.2857	0
	**3**	83.3333	0	66.6666	0	50	0	33.3333	0	16.6666	0	0	0
	**2.5**	80	0	60	0	40	0	20	0	0	0	0	0
	**2**	75	0	50	0	25	0	0	0	0	0	0	0
	**1.5**	66.6666	0	33.3333	0	0	0	0	0	0	0	0	0
	**1**	50	0	0	0	0	0	0	0	0	0	0	0
	**0.5**	0	0	0	0	0	0	0	0	0	0	0	0

### 3.2 Experiment 1b

**FIGURE 1:**
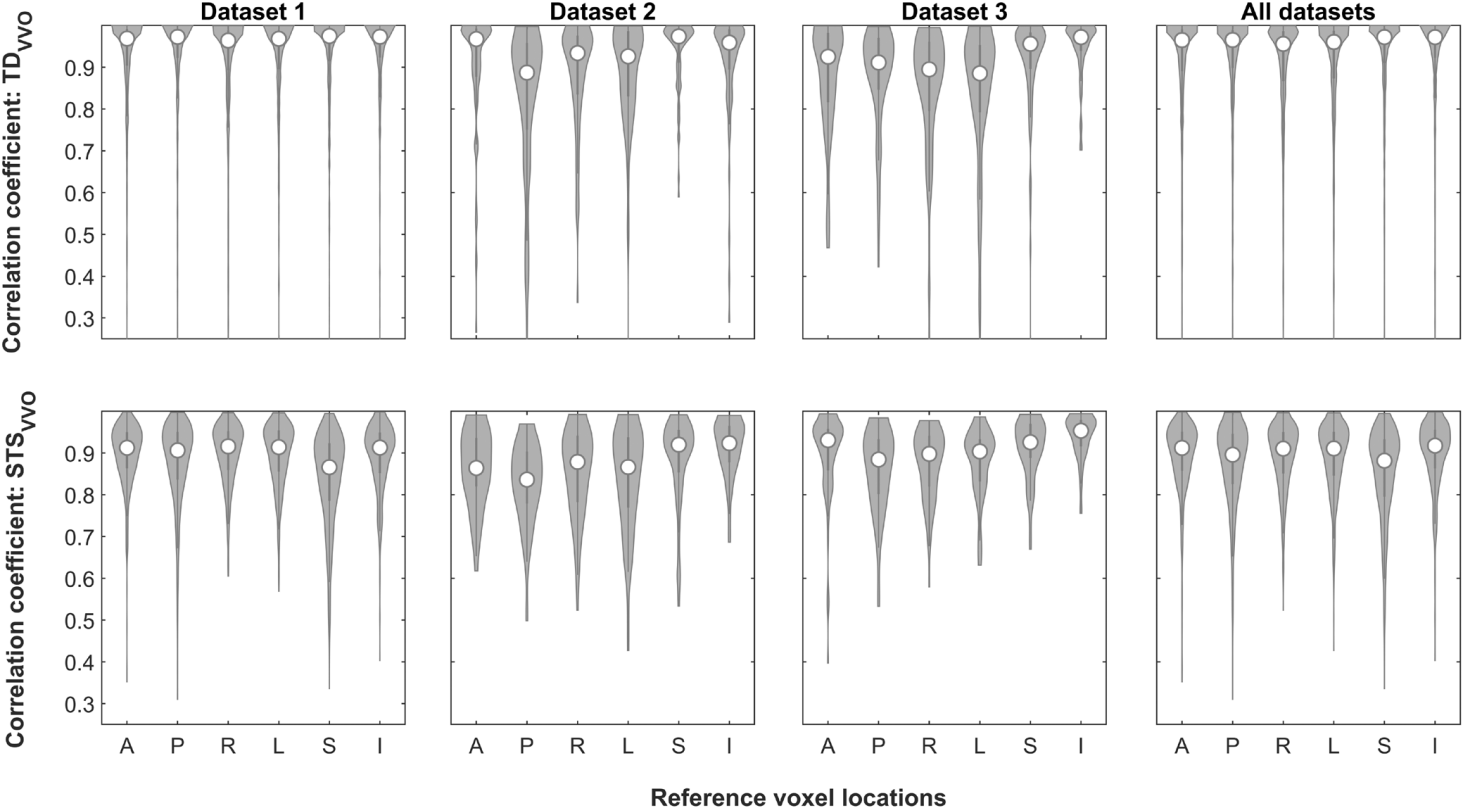
Violin plots of the distribution of the correlations of the six representative voxels with their mean values, for both overlap measures (TD_VVO_, top row, and STS_VVO_, bottom row) in each and all datasets (columns). *A* = anterior; *P* = posterior; *R* = right; *L* = left; *S* = superior; *I* = inferior.

Assessing both overlap measures in the six representative voxels demonstrates substantial variation between voxels in all datasets, confirming the regional heterogeneity of motion effects (Figure 1). The variation is substantially larger in the TD_VVO_ (upper plots) than in the STS_VVO_ measures (lower plots) and larger in the higher‐motion dataset 3. As neither an a priori hypothesis nor a clear pattern was evident arguing for preferring one location over the other, the median TD_VVO_/STS_VVO_ value over all locations was used in all further analyses.

### 3.3 Experiment 1c

**FIGURE 2:**
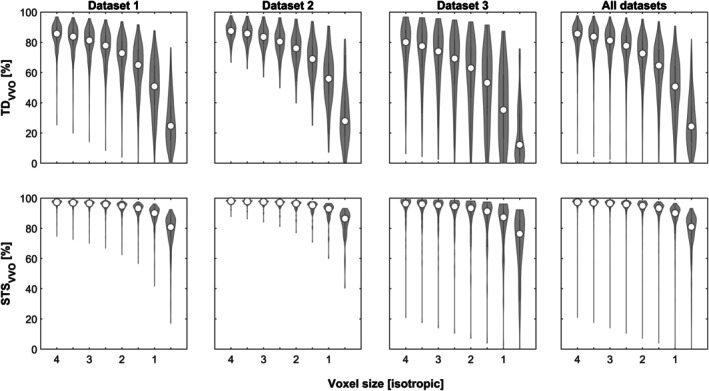
Violin plots of effect of simulated different voxel sizes (X‐axis) on both overlap measures (TD_VVO_, top row, and STS_VVO_, bottom row) in each and all datasets (columns).

Simulating different voxel sizes demonstrates that, for all datasets, both overlap measures show substantial changes as a function of voxel size (Figure 2). The values for TD_VVO_ range from a median of 85.72/87.5/80.11% in the three datasets when simulating isotropic 4 mm voxels, to a median of 24.7/27.87/12.17% when simulating isotropic 0.5 mm voxels. Similarly, the values for STS_VVO_ range from a median of 97.25/98.03/96.38% to a median of 80.79/86.42/76.37%, respectively.

### 3.4 Experiment 1d

**FIGURE 3:**
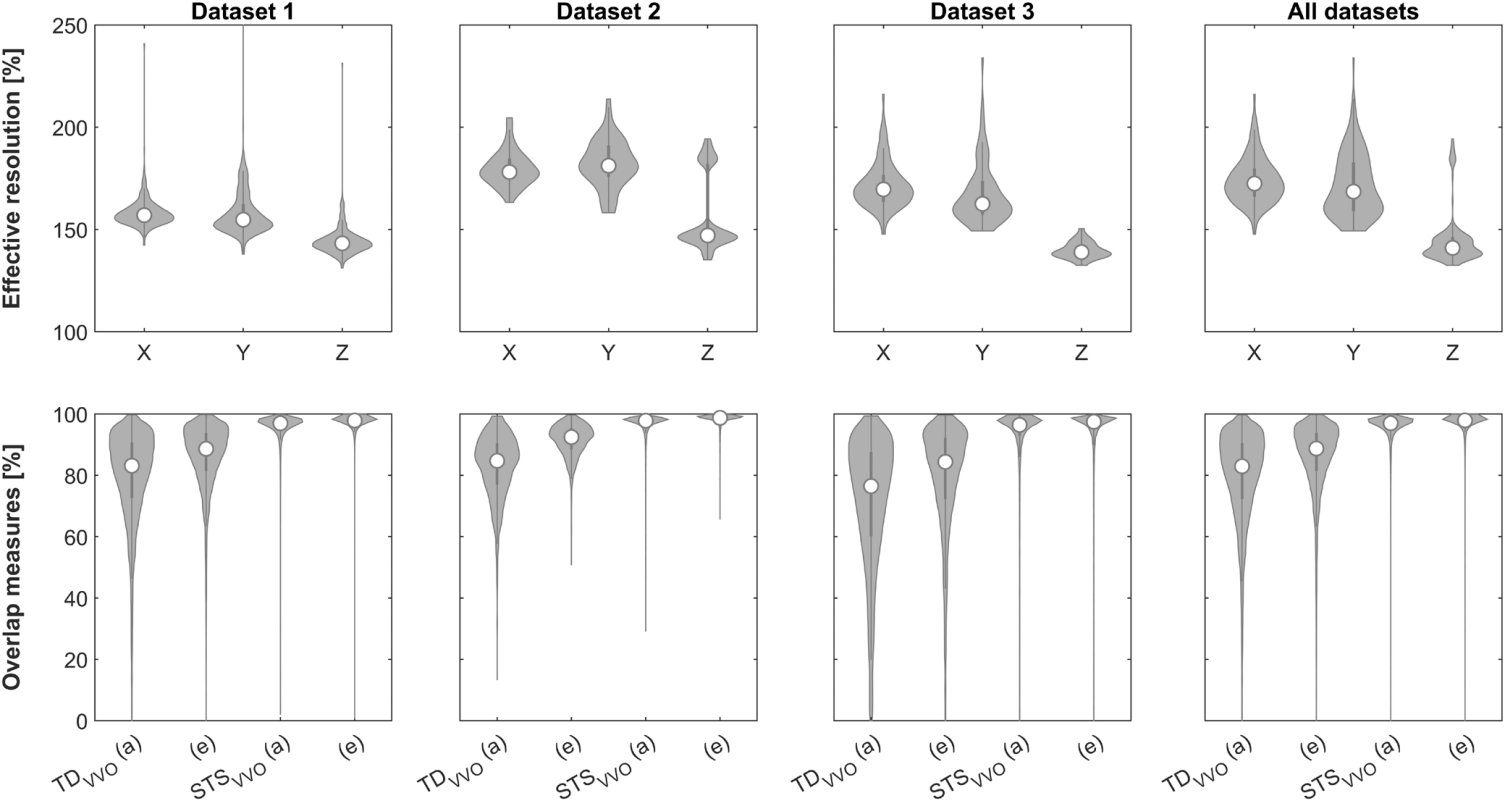
Violin plots of effective voxel sizes in each dimension (X‐axis) in relation to the acquired resolution (=100%; top row), for each and all datasets (columns). Bottom row shows the effect on both overlap measures, in each and all datasets (columns). (a) = acquired, (e) = effective resolution.

Comparing acquired (a) and effective smoothness (e, without any Gaussian smoothing) demonstrates the substantial difference between these two metrics, and consequently a substantial influence on the two overlap measures (Figure 3). Of note, these results also illustrate the effect of the (axial) acquisition as the effective in‐plane resolution (along X and Y) differs substantially from the across‐plane resolution (Z) in each dataset (Mann–Whitney, each *p* ≤ 0.0001, corrected for 6 comparisons).

### 3.5 Experiment 1e

**FIGURE 4:**
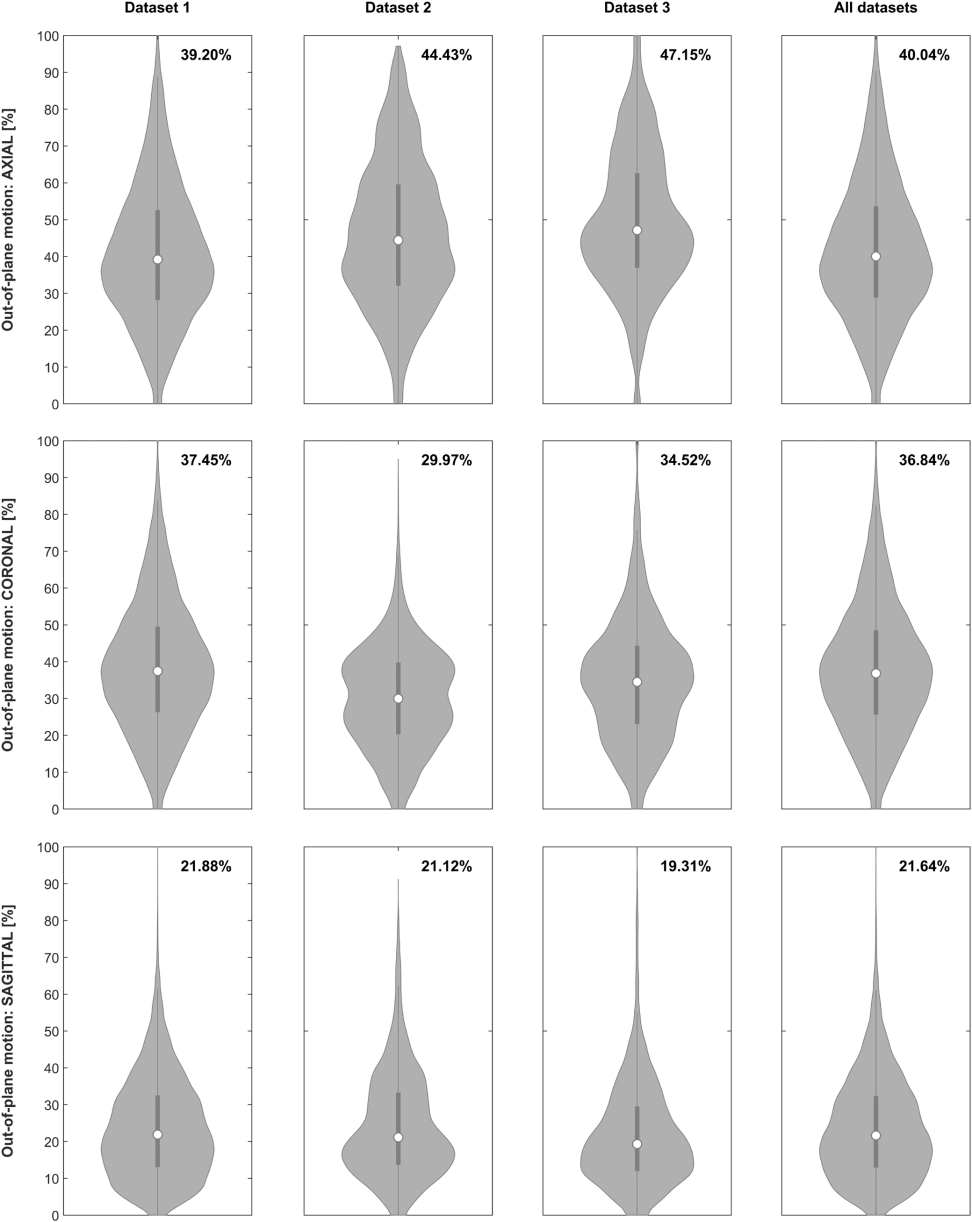
Violin plots of the proportion of out‐of‐plane motion in relation to all motion effects (=100%) in each and all datasets (columns), assuming an axial (top), coronal (middle) or sagittal orientation (bottom row). Note substantially lower median values (inserts) for coronal and sagittal orientation.

Assessing the proportion of out‐of‐plane motion shows that the acquisition plane has a substantial influence (Figure 4). Of note, the highest‐motion dataset 3 has the highest out‐of‐plane movement in the (actual) axial acquisition plane, but would have the lowest such movement if it had been acquired using a sagittal orientation. Also, despite being of very high quality, the proportion of out‐of‐plane movement was higher in the task‐fMRI dataset 2 than in the resting‐state dataset 1.

### 3.6 Experiment 2a

**FIGURE 5:**
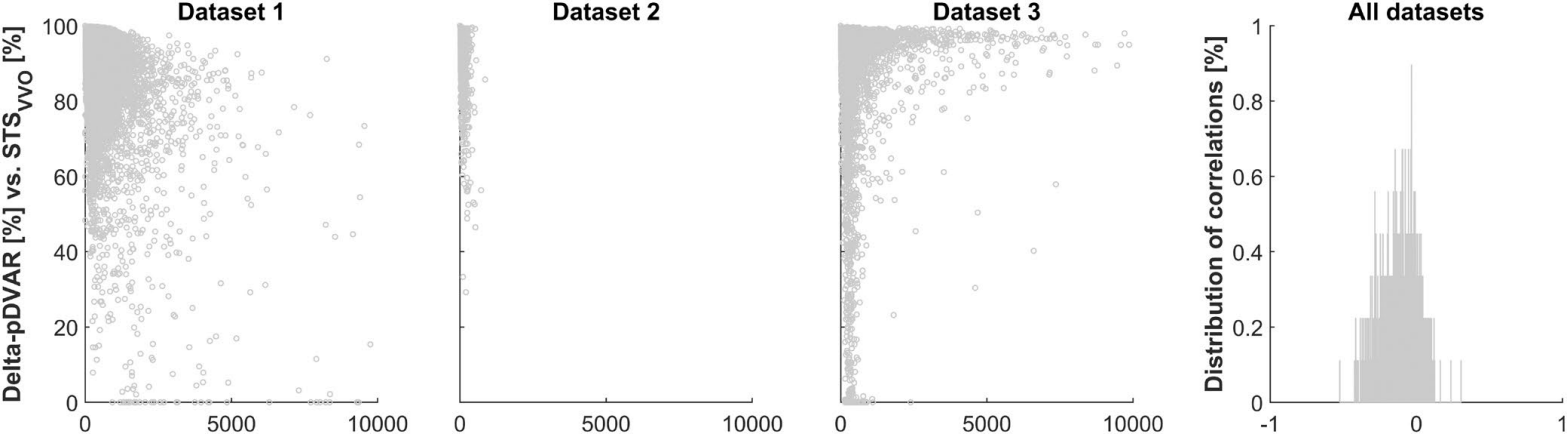
Relation of Δ%D‐VAR with STS_VVO_ in all datasets (right three panels). Also shown is a histogram of all correlation coefficients over all datasets (left panel). Note consistent lack of correlations between both measures.

Comparing STS_VVO_ to Δ%D‐var demonstrates no clear correlation between both measures (Figure 5). This was confirmed by the histogram of the correlation coefficients, the mean of which was only slightly negative across all datasets. Interestingly, the correlation was significant in 58.15% in dataset 1, in 32.5% in dataset 2, but in only 11% in dataset 3.

### 3.7 Experiment 2b

**FIGURE 6:**
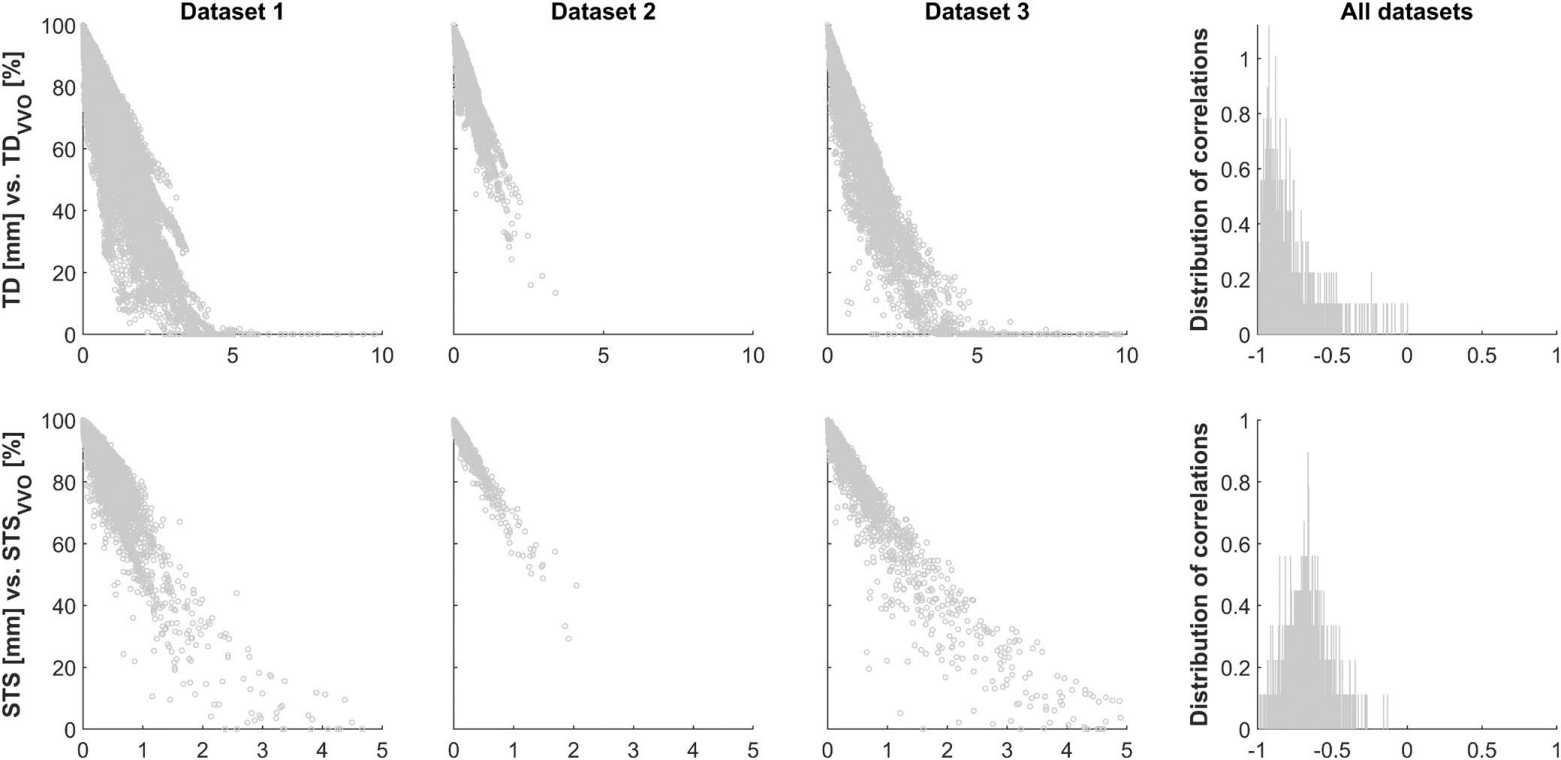
Relation of conventional total displacement (TD, top row, X‐axis) and conventional scan‐to‐scan displacement (STS, bottom row, X‐axis) with both new overlap measures (TD_VVO_, top row, and STS_VVO_, bottom row, Y‐axes), in each dataset (right three columns). Also shown is a histogram of all correlation coefficients between both measures over all datasets (left column). Note strongly negative correlations between TD and TD_VVO_ and weaker negative correlations between STS and STS_VVO_. Also note different scaling of the X‐axis.

Comparing TD_VVO_ and STS_VVO_ to TD and STS demonstrates a high agreement, but also some disagreement between the two parameter pairs (Figure 6). Correlation analyses showed a significant inverse correlation between TD_VVO_ and TD in 99.3/98.75/100% of subjects, and a mean correlation coefficient of *r* = −0.8159, −0.781, and −0.7804 in the three datasets, respectively. Correlating STS_VVO_ and STS showed a significant inverse correlation in 100/100/100% of subjects, and a mean correlation coefficient of *r* = −0.678, −0.6488, and −0.6867 in the three datasets, respectively. While significant in all cases, the correlation itself was weaker for STS_VVO_ and STS than it was for TD_VVO_ and TD.

### 3.8 Experiment 2c

**FIGURE 7:**
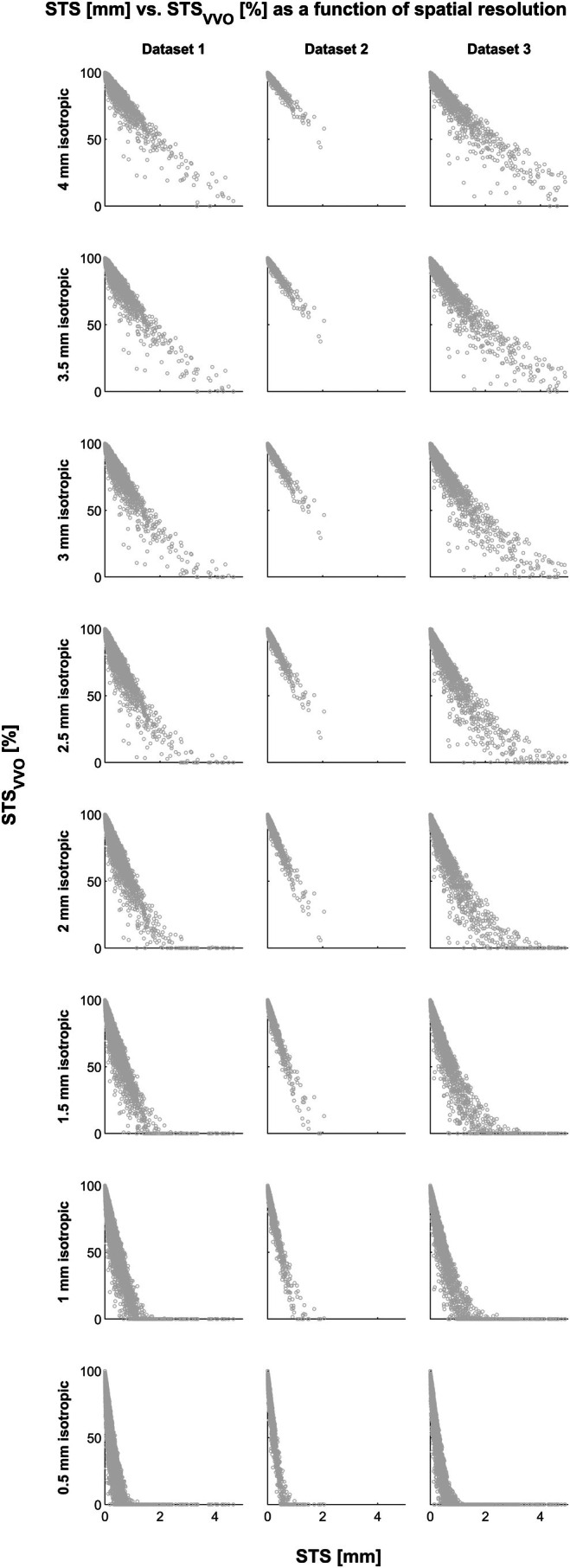
Relation of conventional STS with STS_VVO_ as a function of voxel size (rows), in each dataset (columns). Note consistent change in relation with changes in voxel size.

Further examining the correlation between STS and STS_VVO_ as a function of spatial resolution demonstrates that the overall pattern remains, but the relation changes substantially (Figure 7), from bigger (top) to smaller voxel sizes (bottom).

### 3.9 Experiment 2d

**FIGURE 8:**
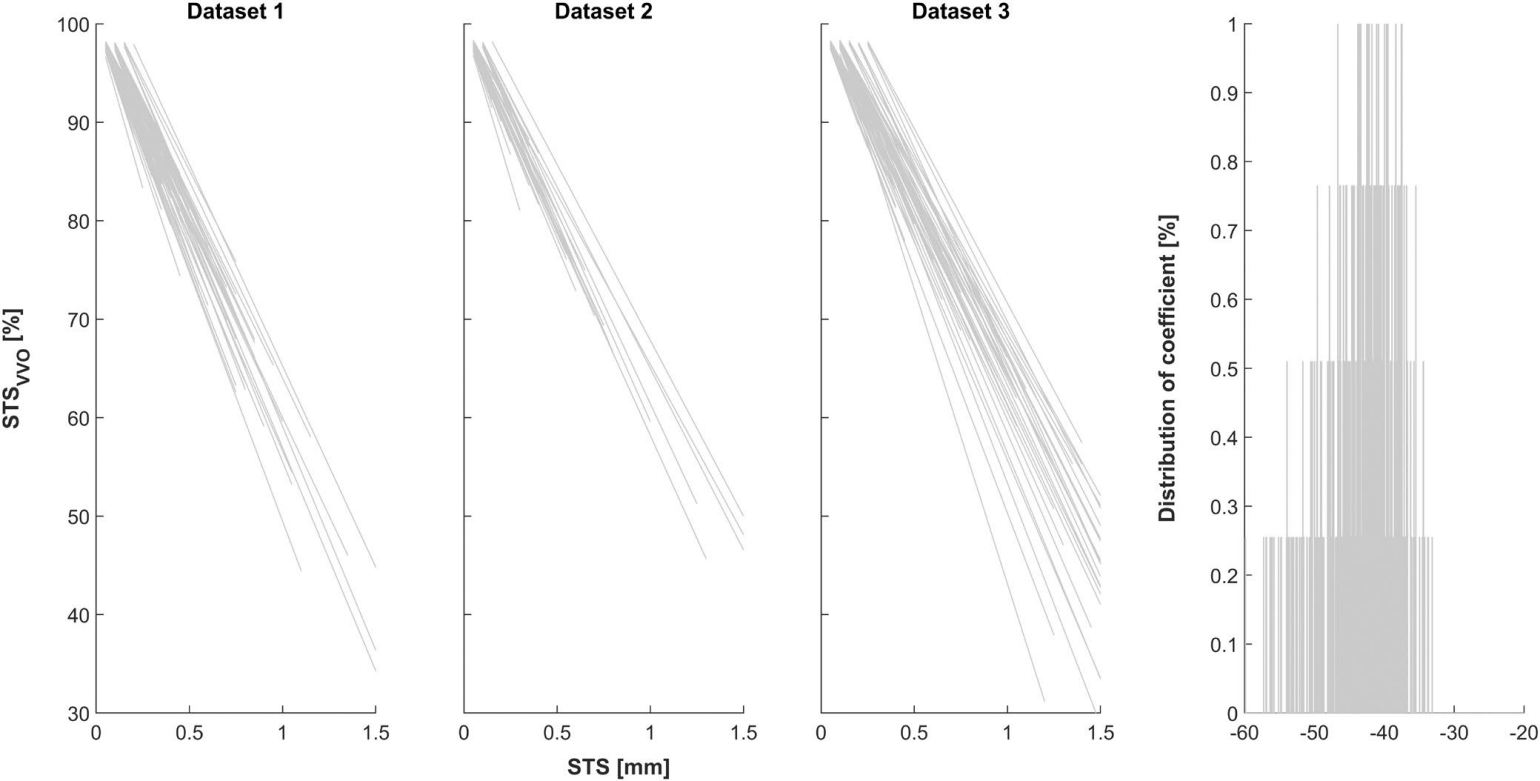
Linear modeling of the relation of STS with STS_VVO_ in a subset of *n* = 392 sessions with identical voxel sizes. Right panels: Histogram of distribution of coefficients (from left panels). See text for details.

When mathematically assessing the individual linear correlation between the two measures in the subset of 392 sessions with identical voxel sizes, a very consistent pattern emerges across datasets (Figure 8). The coefficient *a* obtained from these linear fits was *a* = 43.2422, with a standard deviation of 5.6894. Using this mean coefficient in a linear equation, the corresponding STS/STS_VVO_ values are shown in Table 2.

**TABLE 2:** Illustration of corresponding values of STS (in mm) and STS_VVO_ (in %), derived using linear modelling (cf. Figure 8), for 392 datasets of 3 × 3 × 3 mm acquired resolution. Note that STS_VVO_ values were rounded in the second decimal place. Gray: Exemplary corresponding STS and STS_VVO_ values, see text for details.

STS	STS_VVO_
0	100
0.05	97.84
0.10	95.68
0.15	93.51
0.20	91.35
**0.25**	**89.19**
0.30	87.03
0.35	84.87
0.40	82.70
0.45	80.54
**0.50**	**78.38**
0.55	76.22
0.60	74.05
0.65	71.89
0.70	69.73
0.75	67.57
0.80	65.41
0.85	63.24
0.90	61.08
0.95	58.92
**1.00**	**56.76**
1.05	54.60
1.10	52.43
1.15	50.27
1.20	48.11
1.25	45.95
1.30	43.79
1.35	41.62
1.40	39.46
1.45	37.30
**1.50**	**35.14**

## 4 Discussion

This manuscript was aimed at developing and exploring voxel‐size dependent measures for subject motion in fMRI timeseries.

### 4.1 Experiment 1a

*Validity and accuracy*: the simulations using a known, ground truth amount of motion (Table 1) demonstrate that the algorithm correctly and accurately describes the effect of motion on the volumetric overlap between moved voxels. While only possible for simpler shifts, these results confirm the mathematical validity of the approach and its applicability in this context.

### 4.2 Experiment 1b

*Regional heterogeneity of motion effects*: as expected and as shown previously (Power 2017; Wilke 2012, 2014), motion effects differ substantially between different regions of the brain. In order to be sensitive to this, 6 voxels were systematically placed on the surface of a sphere centered around the image volume's origin. Their correlations with the mean value vary substantially (Figure 1), underlining that it is meaningful to use more than one location to comprehensively describe motion effects. As noted before, the median may be a more robust indicator in such scenarios (Meyer‐Lindenberg et al. 2006) which is why it was also used here to yield a single summary volume overlap indicator per timepoint. Of note, other (or even all) voxel locations can be used, which, naturally, however, substantially prolongs processing times.

### 4.3 Experiment 1c

*Effect of voxel size*: as laid out in the introduction, identical motion must be expected to have dramatically different effects on the smallest spatial component, depending on its size. The effect as demonstrated in theory (cf. Table 1) is also very evident in praxis: from the large (isotropic 4 mm) to the small end of the spectrum (isotropic 0.5 mm) explored here, the median total displacement voxel volume overlap (TD_VVO_) value changes from 85.65% to 24.34%, across all datasets. The scan‐to‐scan voxel volume overlap (STS_VVO_) value does not change as much, from 97.26% to 80.96%, likely reflecting the fact that total displacement is much higher than scan‐to‐scan displacement (across all datasets, mean TD was 0.59 mm, while mean STS was 0.11 mm). The interplay of the extent of motion and voxel size becomes even more evident when comparing the lowest‐ versus highest‐motion quartile of datapoints (supplementary Figure 1). Here, the effect of voxel size clearly is much more dramatic when more motion is present, underlining again that subject motion will have drastically different effects on the overlap between voxels, depending on their size.

### 4.4 Experiment 1d

*Acquired and effective smoothness*: from the results depicted in Figure 3, several conclusions can be drawn. For one, acquired and effective voxel size, as determined by assessing the residuals from the statistical analyses (Kiebel et al. 1999; Worsley 1996) differ substantially not only regionally (Hayasaka et al. 2004) but also as a function of the (here: axial) acquisition plane. Concretely, spatial smoothness is much higher within the acquisition plane than between planes (the impact of this effect on out‐of‐plane motion will be discussed below). Second, these results, in line with the simulations shown in Figure 2, demonstrate that the volume overlap measures are higher when looking at effective resolution (which of course is consequential after having demonstrated that “effective > acquired”). These results could be considered to argue in favor of using the effective resolution instead of the acquired one, as it may more truly reflect the “true” actual voxel size (and thus, volume overlap). However, this measure is only an indirect one, derived from the residuals of a statistical model (Kiebel et al. 1999; Worsley 1996), and is not a known constant (as opposed to the acquired voxel size; Liu et al. 2017). Also, it would then be difficult to argue in how far additional (Gaussian) smoothing may also have to be considered, which is commonly applied during post‐processing. Further, the impact of signal‐to‐nose in the acquired voxel size (Liu et al. 2017; Molloy et al. 2014; Murphy et al. 2007) would not be reflected anymore in such measures, or may be contaminated by post‐processing steps. Hence, it is argued here that the acquired voxel size should still be the “gold standard” reference point for voxel‐size sensitive indicators, although this assessment may change in the future if such measures, and their behavior, are more widely‐used, and more widely‐explored.

### 4.5 Experiment 1e

*In‐* vs. *out‐of‐plane motion*: In the (common) case of an axial acquisition, out‐of‐plane motion corresponds to “above and below” the original acquisition plane, while volume changes induced by shifts in the left–right and anterior–posterior direction are in‐plane. If subject motion was random, out‐of‐plane motion would be expected to be equal in all directions, and hence, independent of the acquisition plane. However, this clearly is not the case: within each and across all datasets, out‐of‐plane motion is higher when assuming an axial, then when assuming a coronal or sagittal orientation (Figure 4). Interestingly, the worse the average data quality is (e.g., dataset 3; cf. Figure 3), the bigger the difference between the highest and the lowest values (assuming an axial vs. a sagittal orientation). This is well in line with nodding head movements being more prominent when more motion is present (Bause et al. 2020; Frew et al. 2022; Zaitsev et al. 2017). Also, the resting‐state dataset 1 has the least amount of out‐of‐plane motion in the axial acquisition, suggesting that such nodding may be more prominent in task‐based fMRI, when subjects “look out” for, e.g., a video screen or instructions and so forth, instead of “resting”. Incidentally, this effect may also explain some of the non‐linear relation between “subject motion” and image quality (Friston et al. 1996; Satterthwaite et al. 2013). Concretely, if more motion is systematically associated with a higher proportion of out‐of‐plane motion (which again is more detrimental to data quality than in‐plane motion [Zaitsev et al. 2017]), these two effects may superimpose, and hence be nonlinearly detrimental. The proportion of out‐of‐plane volume shifts may therefore serve as an additional indicator for data quality, but such recommendations would have to be based on a further exploration of this measure and its properties in various settings.

### 4.6 Experiment 2a

*Correlation with Δ%D‐VAR*. In this experiment, STS_VVO_ was related to the percent change in the magnitude of the fast variance component of DVARS, a quality indicator suggested recently (Δ%D‐VAR; Afyouni and Nichols 2018). However, there was no strong correlation between the two parameters across all datasets (Figure 5), indicating that they reflect different properties. Interestingly, however, the correlation more often was significant in the higher‐quality datasets 1 (~one in two) and 2 (~one in three), but in only one in 9 in the low‐quality dataset 3, which seems to suggest that “milder” motion is more closely related to the signal changes underlying Δ%D‐VAR. Another reason for the lack of agreement may be that the actual change in the signal is naturally a consequence of many factors (Godenschweger et al. 2016; Liu 2016; Murphy et al. 2013; Zaitsev et al. 2015, 2017) while STS_VVO_ “only” reflects the volumetric effects of subject motion. Analyzing datasets for which more information (such as accompanying data on technical [gradient warming] and physiological factors [heart rate, breathing etc.]); (Bianciardi et al. 2009; Chang and Glover 2009; Glover et al. 2000; Hutton et al. 2011) may help to shed further light on which factors influence Δ%D‐VAR, but not STS_VVO_, in the future.

### 4.7 Experiment 2b

*Relation with conventional measures of subject motion*. Comparing the commonly‐used measures of total displacement and scan‐to‐scan displacement (TD & STS; Power et al. 2012; Wilke 2014) with the here‐suggested volume‐overlap counterparts (TD_VVO_ & STS_VVO_) reveals that they are highly correlated (Figure 6). As expected, the correlation is negative, as higher values for translation or rotation will usually result in lower volume overlap. Interestingly, the correlation again is stronger for the TD/TD_VVO_ comparison, and again, the absolute values are substantially higher for TD (note different scaling of the X‐axis). This confirms that the new parameters show a strong correlation with the established parameters, which is reassuring. However, it also demonstrates that they are by no means mutually interchangeable: the same scan‐to‐scan displacement may induce a very different volume overlap parameter. For example, the STS value of 1 mm may correspond to STS_VVO_ values of 43.75% to 79.64%, to 57.01% to 70.57%, or to 22.1% to 71.97% in the three datasets, respectively. The reason for this may be that STS, as well as TD, is a summarizing indicator, integrating all six parameters describing subject motion into one value (Wilke 2014). In contrast, to yield STS_VVO_, all parameters are applied in full and only their combined volumetric effect is then summarized.

### 4.8 Experiment 2c

*Impact of voxel size on the relation with conventional measures of subject motion*. Extending the results from Figure 5, the more relevant parameters pair (STS and STS_VVO_) was plotted against each other, assuming the 8 different simulated voxel sizes already used for Experiment 1c (cf. Figure 2). Very clearly, the patterns remain similar (Figure 7), but their numeric correspondences change drastically as a function of voxel size. This again demonstrates that identical subject motion will have very different effects on the volume overlap between (in this case, subsequent) voxels, depending on their size.

### 4.9 Experiment 2d

*Generalizable defaults*: Coming up with default values for a new approach is always difficult. This is also the case here, as existing approaches either do not account for voxel size (TD, STS) or show no clear correlation with the new approach (Δ%D‐var); consequently, they cannot serve as a gold standard reference. We therefore assessed a homogeneous subset of 392 sessions from all datasets (with identical voxel sizes and a total of 46.262 datapoints) with respect to the individual correlation between STS_VVO_ and STS thresholds (Figure 8), ultimately using this data to mathematically derive their interrelation across subjects. This again allows us to generate corresponding values for this scenario (Table 2). As shown above (cf. Figure 7), STS will have different effects in datasets of a different voxel size, but in contrast to this, the values of STS_VVO_ can be generalized from this table to be applicable to other datasets, irrespective of their resolution. This allows for a meaningful comparison of motion effects in datasets of inhomogeneous voxel sizes (particularly in multisite studies; Dansereau et al. 2017; Reardon et al. 2021). As can be seen in Table 2, the STS_VVO_ values corresponding to STS thresholds of 0.25, 0.5, 1, and 1.5 mm are close to 90%, 80%, 55%, and 35%, respectively, when assessing the acquired resolution. Hence, it is pragmatically suggested that an STS_VVO_ cutoff value of 90% can be considered very strict, and 80% as strict. Values below that, but above 55% must be considered lenient, and 35% very lenient. Datapoints with STS_VVO_ values above 90% could consequently be considered very high quality, those above 80% as high quality, and those above 55% as only potentially acceptable. Values below 55% must be considered questionable. These values are therefore used as defaults in the algorithm, although these settings can easily be adapted.

### 4.10 Possible Limitations of This Study

Motion is only one of several factors that introduce variance, among them physiological and technical factors (heart beat, breathing, gradient warming/scanner drifts, etc.; Bianciardi et al. 2009; Chang and Glover 2009; Glover et al. 2000; Hutton et al. 2011) as well as spin‐history effects and motion during data acquisition (Drobnjak et al. 2006; Lee et al. 1998). No one measure can therefore capture all relevant sources of data contamination, which is also true for the overlap measures proposed here.

Also, while the STS defaults explored in Experiment 2d are in line with previously‐used thresholds (Parkes et al. 2018; Power et al. 2012; Wilke and Baldeweg 2019), others may be used, even outside the range depicted in Table 2. To this effect, however, the coefficient *a* as detailed in the results section may be used, and the approach itself, of course, is generic and can be used in new samples to validate and/or extend the results presented here.

### 4.11 Implementation and Availability

The code developed for this work is freely available as a toolbox for SPM, running within Matlab, and can therefore be seamlessly integrated into existing data processing streams. With the defaults mentioned above (mainly “using six exemplary voxel locations”, which is the time‐limiting setting), it typically requires less than 1 min to run on an average fMRI dataset of a few hundred volumes.

### 4.12 Real‐World Applications

The algorithm comes with a number of possible real‐world applications. For one, it is targeted toward researchers working with higher‐resolution fMRI data where the issue of progressively smaller voxel size makes determining appropriate motion thresholds more pressing. Whether in this context in particular, using effective as opposed to acquired resolution is warranted will require further investigations (hence, the functionality to assess effective resolution of a dataset “on the fly” is included, but not used by default).

For fMRI sequence programmers and ‐optimizers alike, the option to assess the exact proportion of motion‐induced out‐of‐plane volume changes offers new opportunities to deal with these effects by adjusting acquisition parameters. For example, while more data is needed before generally recommending sagittal acquisition for task‐based fMRI (which leads to the typical “nodding” motions to at least result in in‐plane, instead of out‐of‐plane motion), the algorithm now allows for just these studies.

Also, clinical or neuroscientific studies using, for example, heterogeneous clinical or multisite fMRI data are now able to apply a common quality control threshold, irrespective of differences in data resolution. This allows for the easier pooling of datasets from, for example, rare disorders, taking this confound into account (cf. the graphical abstract, where “high quality” data turns into “acceptable” solely as a function of voxel size).

The effect of using either new parameter as covariates to explain motion‐related variance (Friston et al. 1996; Wilke 2014) has not been explored here (partly due to a multitude of possible combinations, including shifted and squared versions; Wilke 2012). Of note, the option to derive the voxel volume overlap parameters for each individual voxel (see also visualizations, below) may be of interest in this context, potentially allowing for voxelwise covariates in statistical analyses. If requested, the toolbox will also generate a regressor of volumes falling below a given VVO threshold which may then be used for censoring/scrubbing, akin to previous approaches (Lemieux et al. 2007; Power et al. 2014; Satterthwaite et al. 2013; Wilke and Baldeweg 2019). This approach may be particularly relevant in clinical populations which may move more, but where every datapoint is precious (Wilke et al. 2018).

Finally, the toolbox allows for an extensive visual exploration of motion effects over space and time. Routinely, it generates a comprehensive two‐part graphical output (see Supplementary Figure 2 for some representative [good, typical, bad] examples) and stores all relevant results in a mat‐file. Additionally, all regions within a standard atlas can be explored, and the evolution of TD_VVO_ and STS_VVO_ over time can be visualized and explored for (the center of) each region (see Supplementary Figure 3 [same cases as above]). Finally, all voxels in a slice can be assessed, allowing for the visualization of the temporal as well as spatial characteristics of the two overlap measures in the form of a video (see Supporting Information [Link], [Link], and 3 [same cases as above]).

### 4.13 Summary and Outlook

This work proposes, evaluates, and explores two new measures for subject motion in functional MRI, which are sensitive to the acquired voxel size. The new features' properties are explored in and across three large datasets with different motion characteristics, and meaningful defaults for its interpretation are derived by mathematical modeling. The approach also allows for assessing the proportion of out‐of‐plane motion, which may yield important information hitherto unavailable for study. It is made available to the scientific community in the hope that it may prove useful as a generalizable indicator to compare motion across subjects.

## Conflicts of Interest

The author declares no conflicts of interest.

## Supporting information


**Supplementary Figure 1** Extending the results from Figure 2, assessing the lowest and the highest quartile of subject motion illustrates the interplay between voxel size and subject motion for both overlap measures (TD_VVO_, top row, and STS_VVO_, bottom row) in each and all datasets (columns).

**Supplementary Figure 2** Representative examples of actual algorithm outputs for 3 subjects with very little or little motion (“good”, pages 1–2 and “typical”, pages 3–4) and with excessive motion (“bad”, pages 5–6). See also corresponding supplementary Figure 3 and supplementary video material 1, 2, and 3.

**Supplementary Figure 3** Representative examples of TD_VVO_ and STS_VVO_ for all regions in a standard neuroanatomical atlas (Neuromorphometrics atlas [www.neuromorphometrics.com] included with SPM), from the same “good”, “typical” or “bad” subjects shown in Supplementary Figure 2 and supplementary video material 1, 2, and 3. Note discrepancy between slow drifts (more notable in TD_VVO_) and fast motion (more notable in STS_VVO_). Also note spatial heterogeneity between regions (*Y*‐axis), again more obvious for TD_VVO_.

**Supporting Information 1** Video of the two overlap measures over time in an axial slice (20 of 40) from a “good” subject (cf. pages 1–2 in Supplementary Figures 2 and 3). Note some slow drifts, leading to TD_VVO_ falling below 80% (orange) as of frame 31, and illustrative demonstration of spatially heterogeneous effects of subject motion (lower left panel, e.g., frame 65), but consistently excellent STS_VVO_ values (lower right panel) over all frames.

**Supporting Information 2** Video of the two overlap measures over time in an axial slice (24 of 47) from a “typical” subject (cf. pages 3–4 in Supplementary Figures 2 and 3). Note overall more pronounced drifts in TD_VVO_ (lower left panel) and some quick motion spikes leading to lower STS_VVO_ values (lower right panel, e.g., frames 14 and 18). Also note discrepancy between consistently “red” values in TD_VVO_ (values < 55%), but “green” values (values > 90%) in STS_VVO_, suggesting a gradual shift away from the original position.

**Supporting Information 3** Video of the two overlap measures over time in an axial slice (20 of 40) from a “bad” subject (cf. pages 5–6 in Supplementary Figures 2 and 3). Note almost immediate onset of very strong motion leading to very low values for TD_VVO_ as well as STS_VVO_ across practically all frames. Again note spatially heterogeneous motion effects due to complex head movements for both parameters.

**Supporting Information 4** Documentation of all individual subjects contained in datasets 1, 2, and 3.

## Data Availability

The code to generate the measures described in this manuscript is available for free download as a script running in Matlab, relying on functions available in SPM12/25 (Wellcome Department of Imaging Neuroscience) at our group's website at https://www.medizin.uni‐tuebingen.de/de/das‐klinikum/einrichtungen/kliniken/kinderklinik/forschung/forschung‐iii/software# (short link: https://t1p.de/EPN‐software).
