# Supplementary figures and images for: Voxel Volume Overlap: Voxel‐Size Sensitive Indicators of Subject Motion in Functional MRI
Source: Hum Brain Mapp. 2025 Sep 9;46(13):e70337. doi: 10.1002/hbm.70337 (PMC12418571; doi:10.1002/hbm.70337)

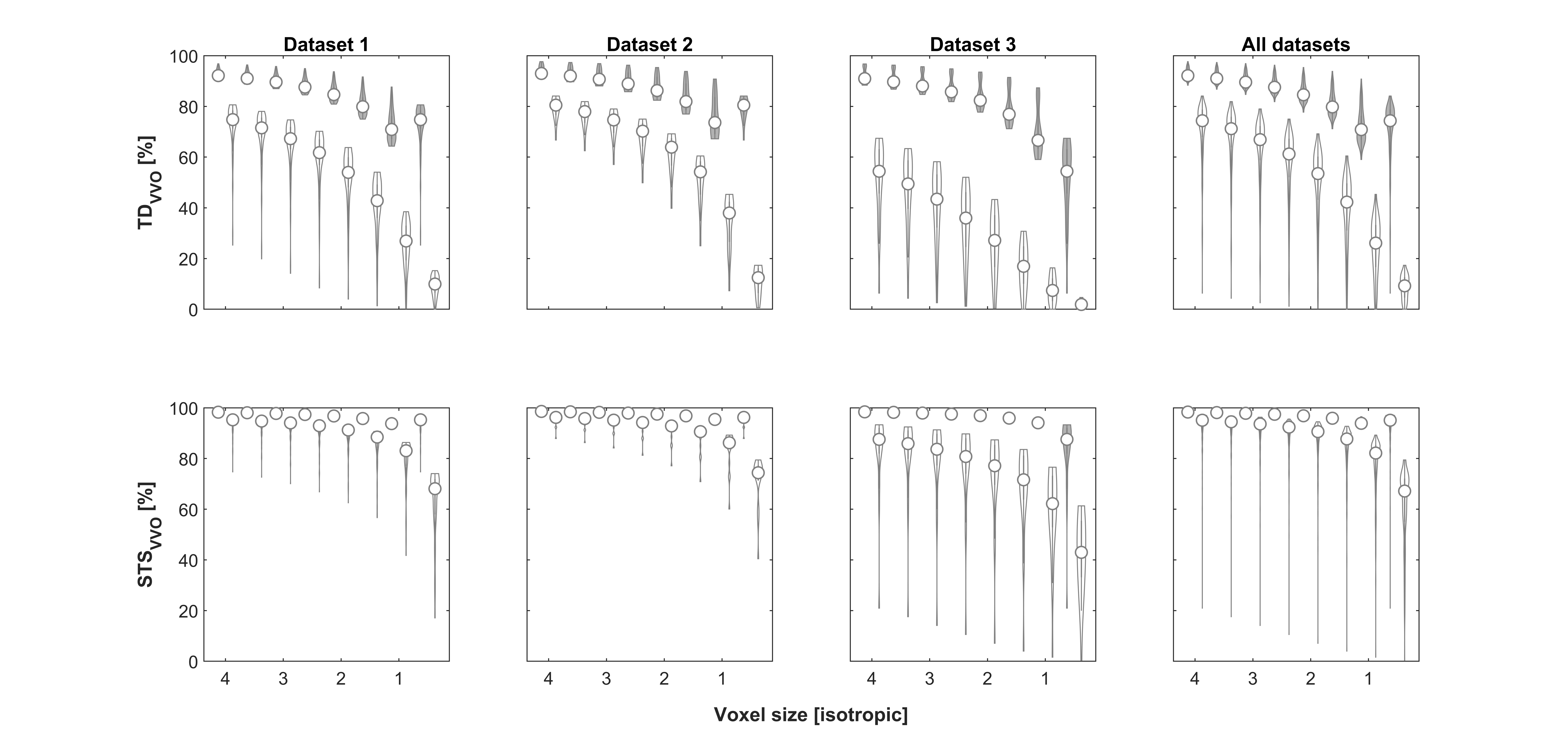

Supplement: Supplementary file 1 — Supplementary Figure 1 Extending the results from Figure 2, assessing the lowest and the highest quartile of subject motion illustrates the interplay between voxel size and subject motion for both overlap measures (TDVVO, top row, and STSVVO, bottom row) in each and all datasets (columns). [file HBM-46-e70337-s006.png]
