# Supplementary material for: Voxel Volume Overlap: Voxel‐Size Sensitive Indicators of Subject Motion in Functional MRI
Source: Hum Brain Mapp. 2025 Sep 9;46(13):e70337. doi: 10.1002/hbm.70337 (PMC12418571; doi:10.1002/hbm.70337)
Supplement: Supplementary file 2 — Supplementary Figure 2 Representative examples of actual algorithm outputs for 3 subjects with very little or little motion (“good”, pages 1–2 and “typical”, pages 3–4) and with excessive motion (“bad”, pages 5–6). See also corresponding supplementary Figure 3 and supplementary video material 1, 2, and 3. [file HBM-46-e70337-s001.pdf]

Case 1 - "good"

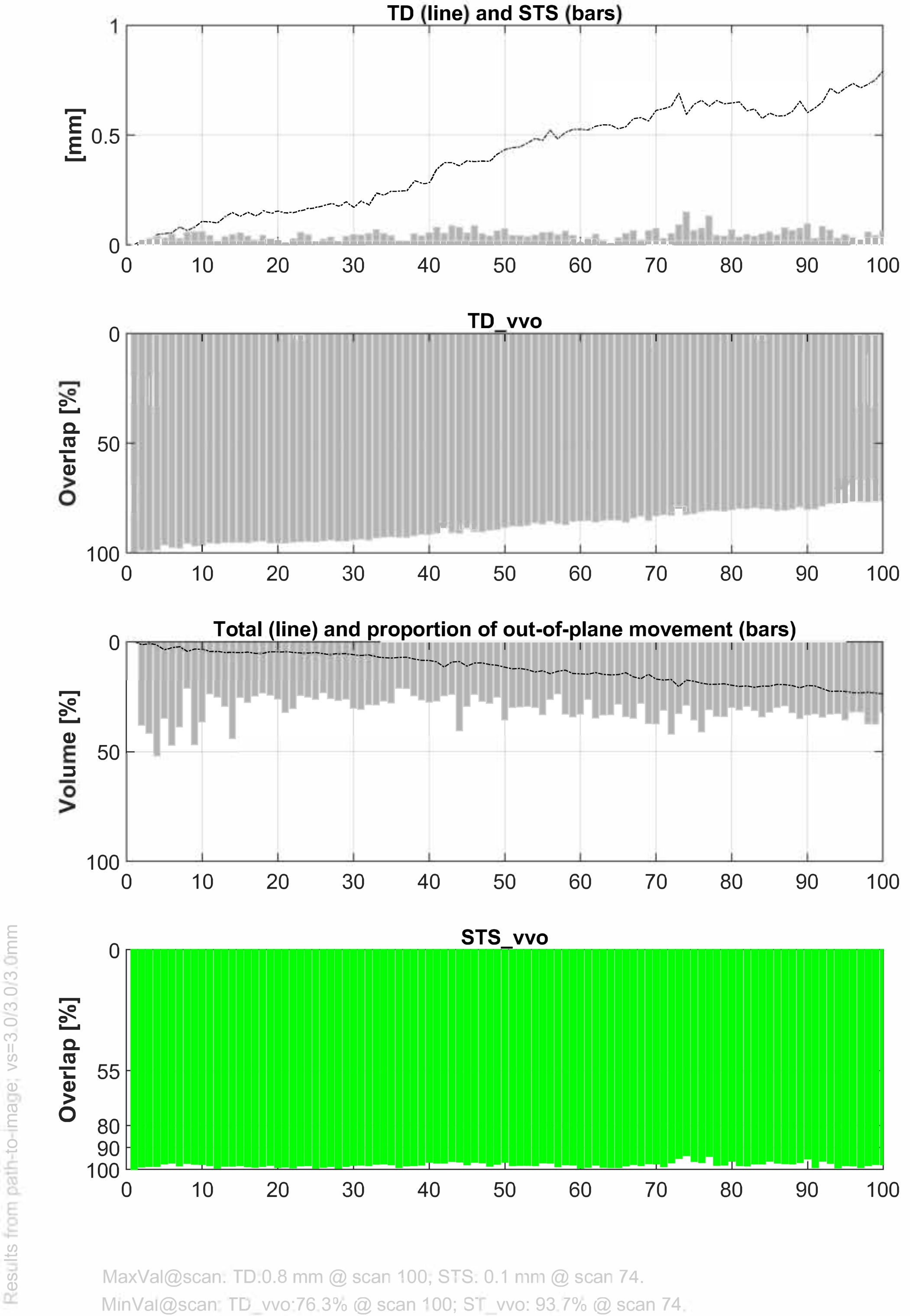
TD (line) and STS (bars)
[mm]
TD_vvo
Overlap [%]
Total (line) and proportion of out-of-plane movement (bars)
Volume [%]
STS_vvo
Overlap [%]
Results from path-to-image; vs=3.0/3.0/3.0mm
MaxVal@scan: TD:0.8 mm @ scan 100; STS: 0.1 mm @ scan 74.
MinVal@scan: TD_vvo:76.3% @ scan 100; ST_vvo: 93.7% @ scan 74.

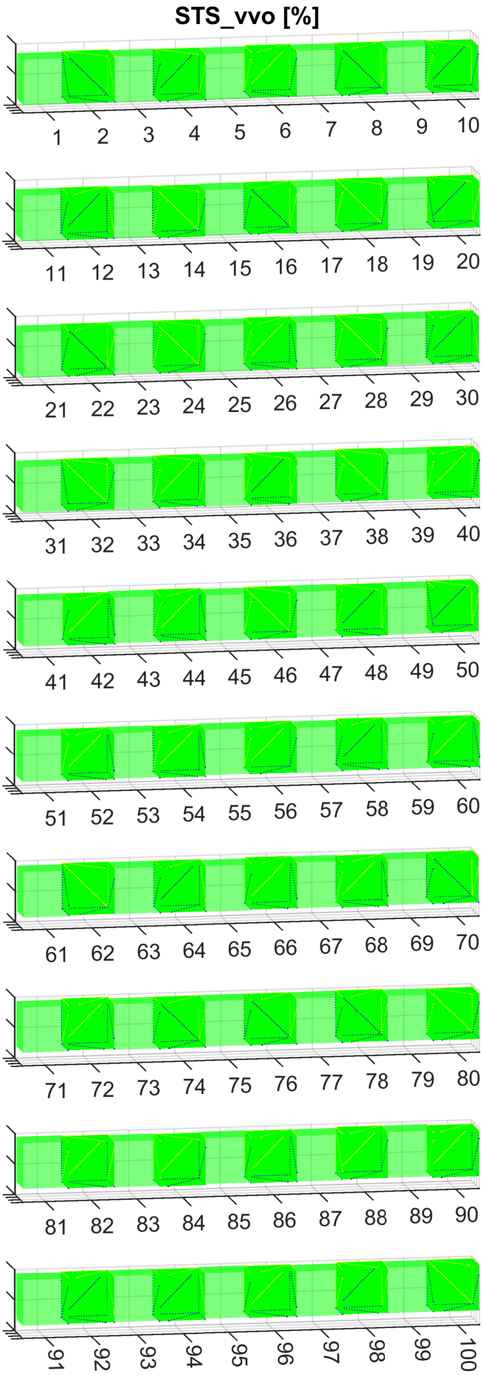

Results from path-to-image; vs=3.0/3.0/3.0mm

MaxVal@scan: TD:0.8 mm @ scan 100; STS: 0.1 mm @ scan 74.

MinVal@scan: TD_vvo:76.3% @ scan 100; STS_vvo: 93.7% @ scan 74.

Case 2 - "typical"

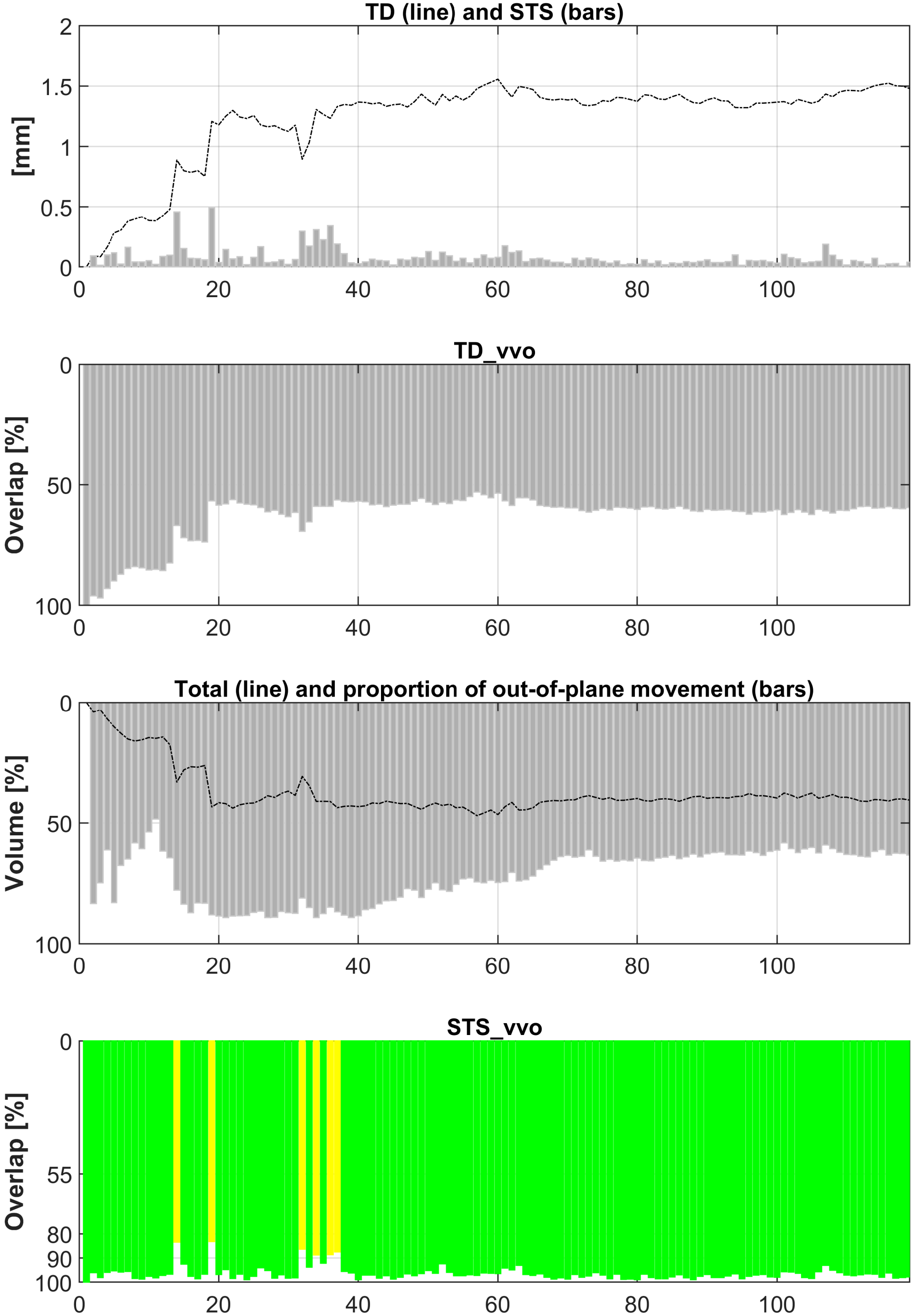

Results from path-to-image; vs=3.0/3.0/3.0mm

MaxVal@scan: TD:1.6 mm @ scan 60; STS: 0.5 mm @ scan 19.

MinVal@scan: TD_vvo:53.0% @ scan 57; ST_vvo: 83.1% @ scan 19.

STS_vvo [%]

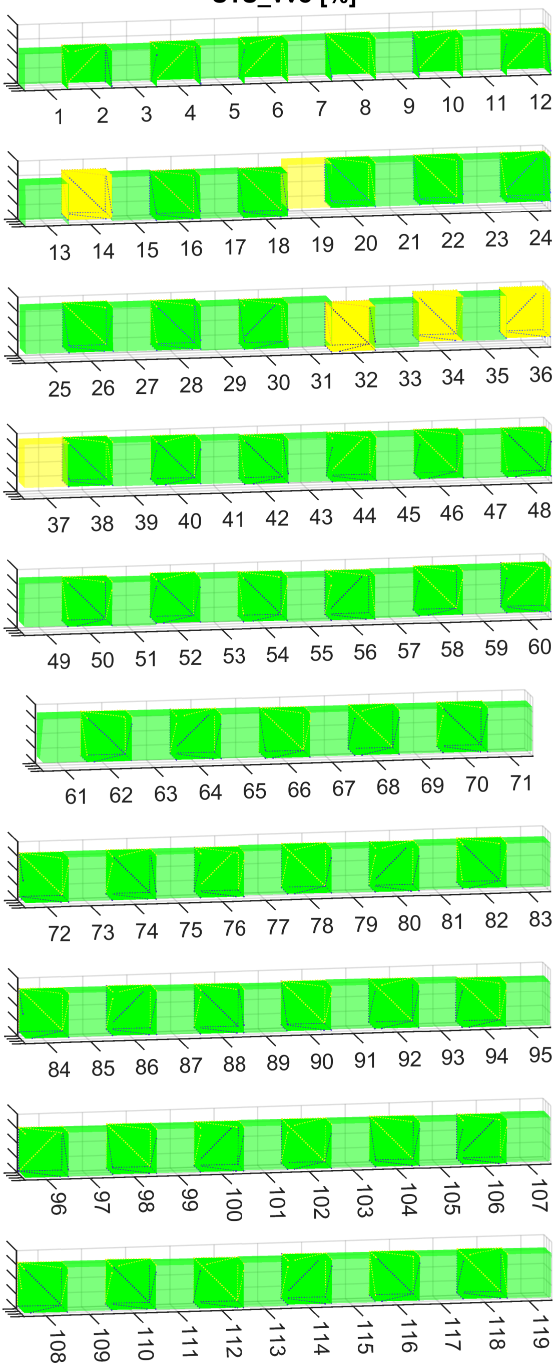

Results from path-to-image; vs=3.0/3.0/3.0mm

MaxVal@scan: TD:1.6 mm @ scan 60; STS: 0.5 mm @ scan 19.

MinVal@scan: TD_vvo:53.0% @ scan 57; STS_vvo: 83.1% @ scan 19.

Case 3 - "bad"

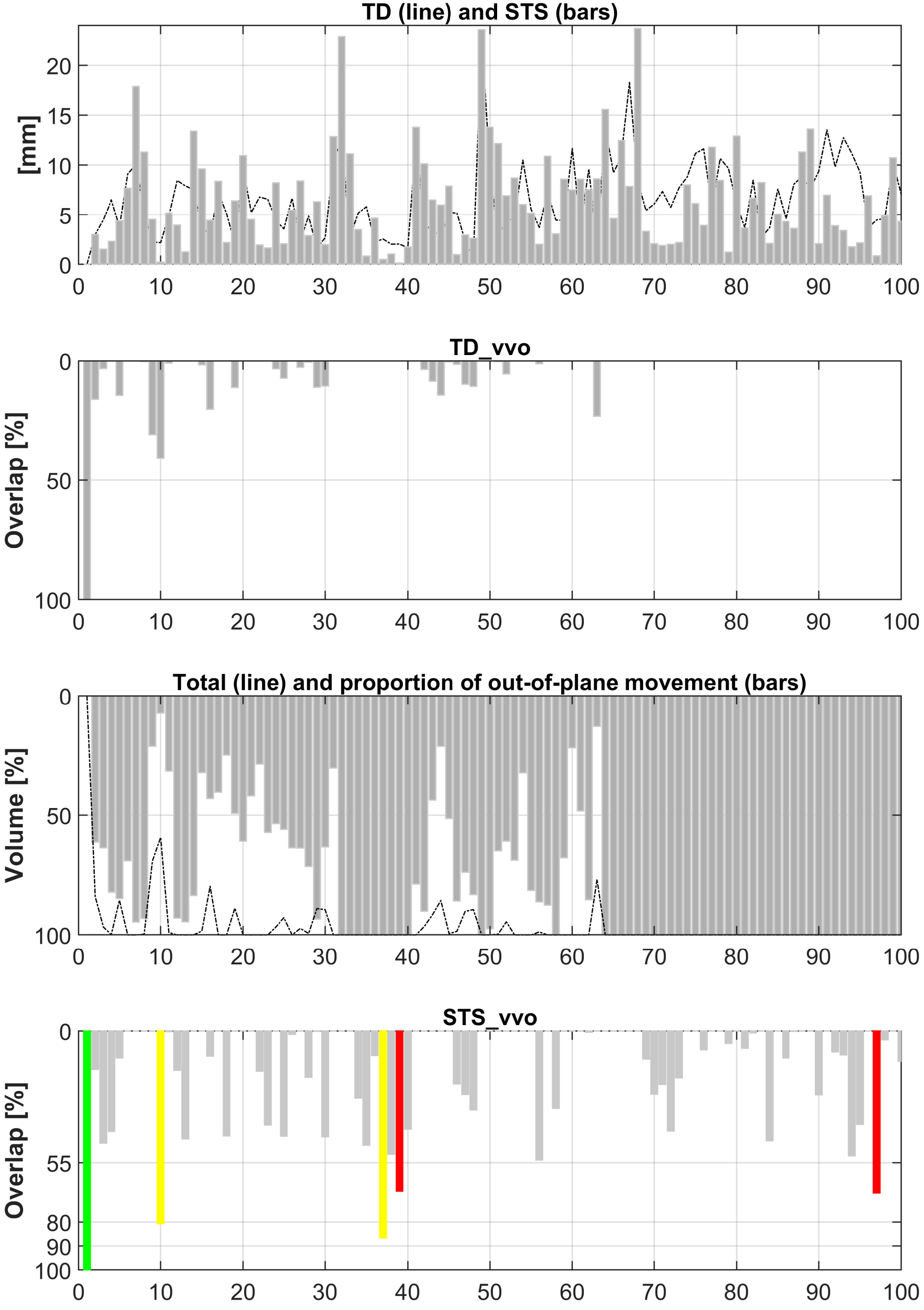

Results from path-to-image; vs=3.0/3.0/3.0mm

MaxVal@scan: TD:23.2 mm @ scan 49; STS: 23.7 mm @ scan 68.

MinVal@scan: TD_vvo:0.0% @ scan 4; ST_vvo: 0.0% @ scan 6.

STS_vvo [%]

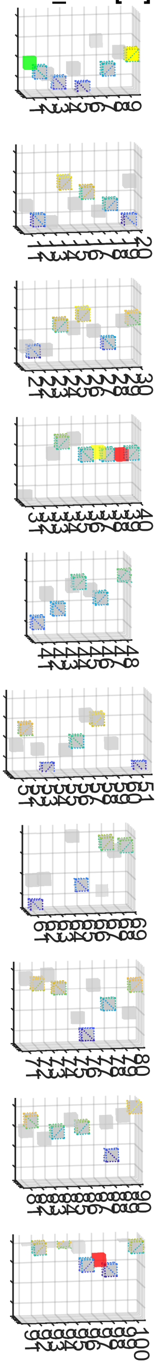

MaxVal@scan: TD:23.2 mm @ scan 49; STS: 23.7 mm @ scan 68.

MinVal@scan: TD_vvo:0.0% @ scan 4; STS_vvo: 0.0% @ scan 6.

Results from path-to-image; vs=3.0/3.0/3.0mm
