# Supplementary material for: Voxel Volume Overlap: Voxel‐Size Sensitive Indicators of Subject Motion in Functional MRI
Source: Hum Brain Mapp. 2025 Sep 9;46(13):e70337. doi: 10.1002/hbm.70337 (PMC12418571; doi:10.1002/hbm.70337)

TD_OV in 131 regions of labels_Neuromorphometrics
(subject "good"; > 90%: green; > 80%: yellow; > 55%: red; < 55%: gray)

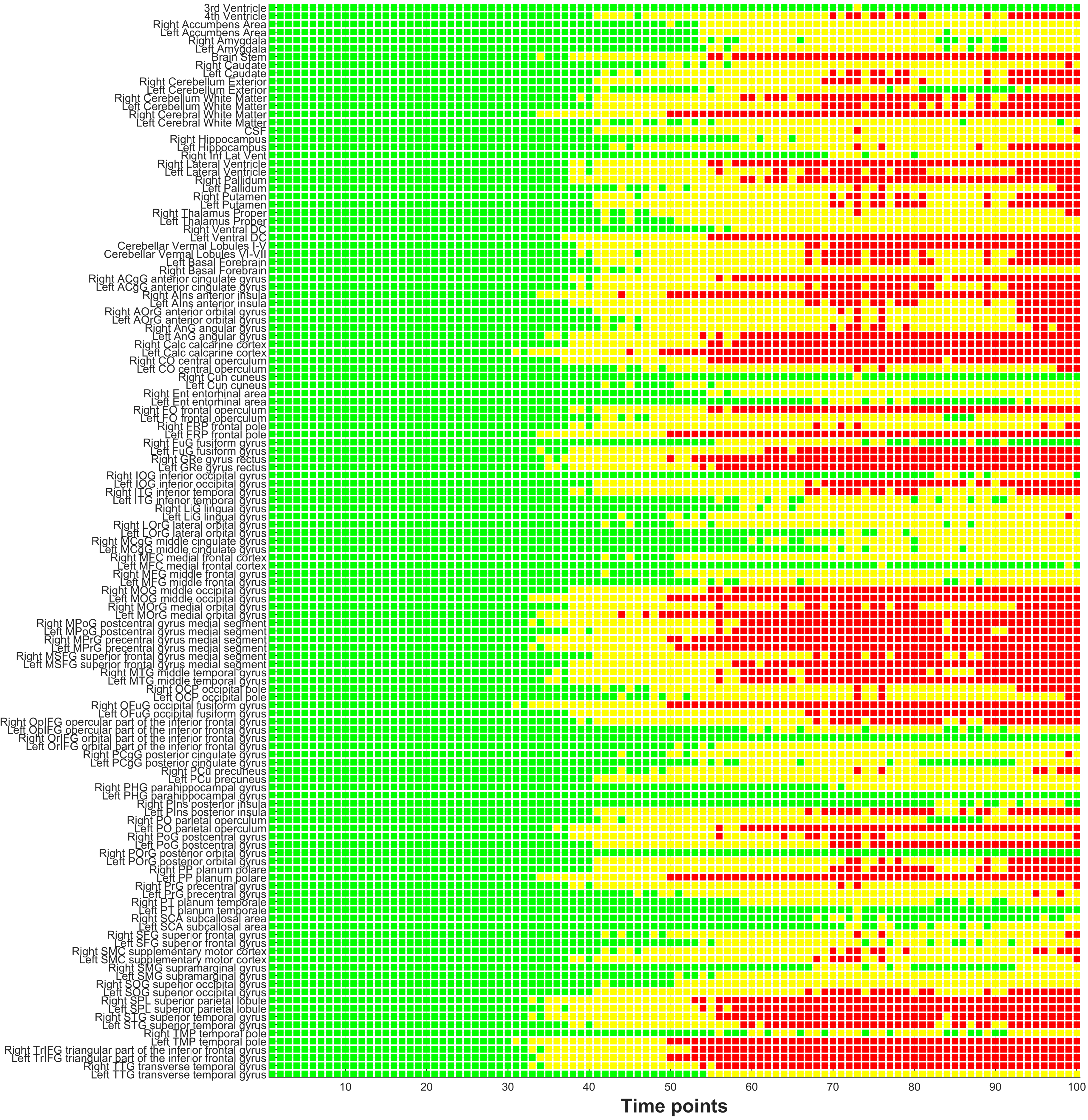

STS_OV in 131 regions of labels_Neuromorphometrics
(subject "good"; > 90%: green; > 80%: yellow; > 55%: red; < 55%: gray)

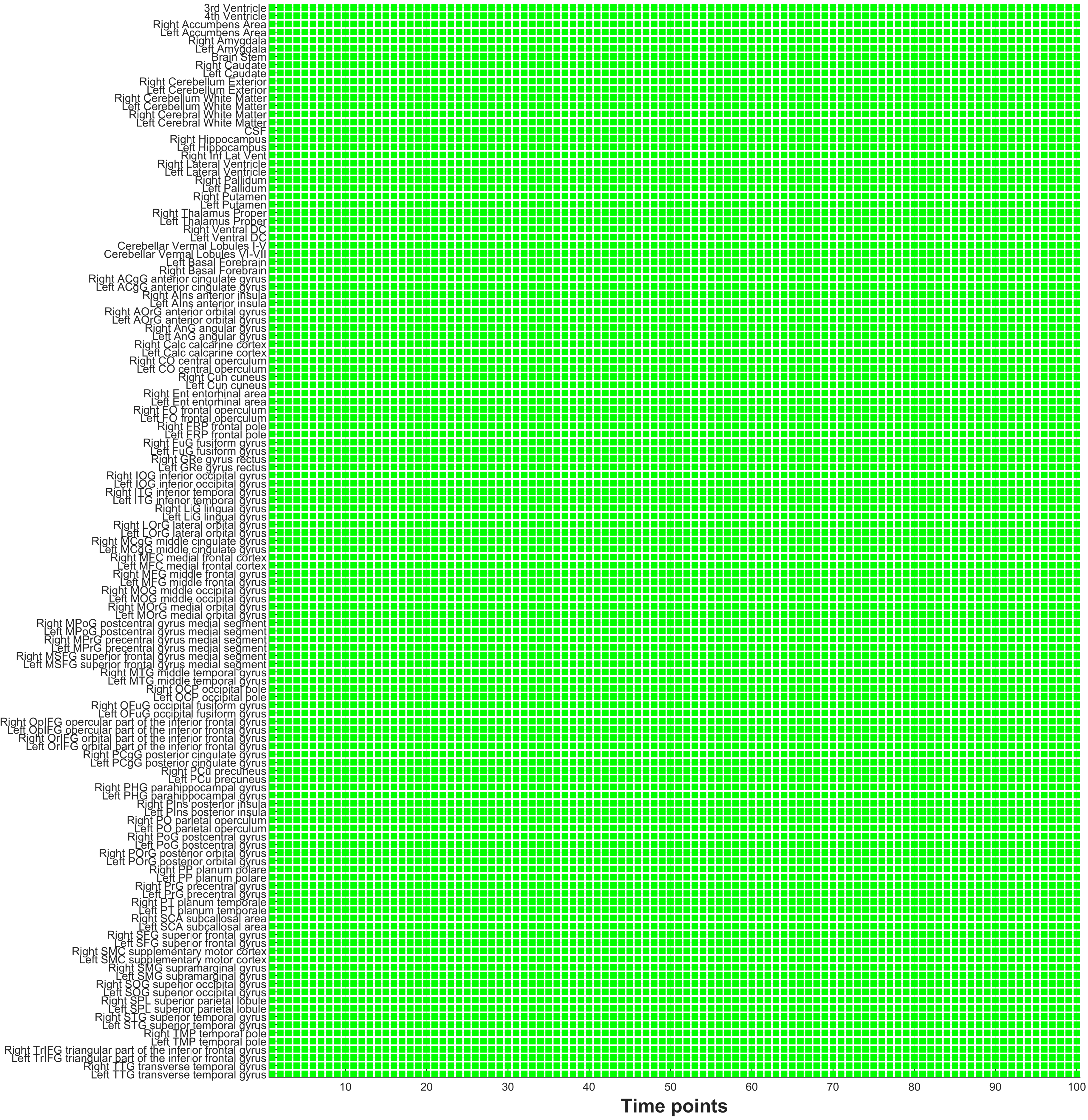

TD_OV in 129 regions of labels_Neuromorphometrics
(subject "typical"; > 90%: green; > 80%: yellow; > 55%: red; < 55%: gray)

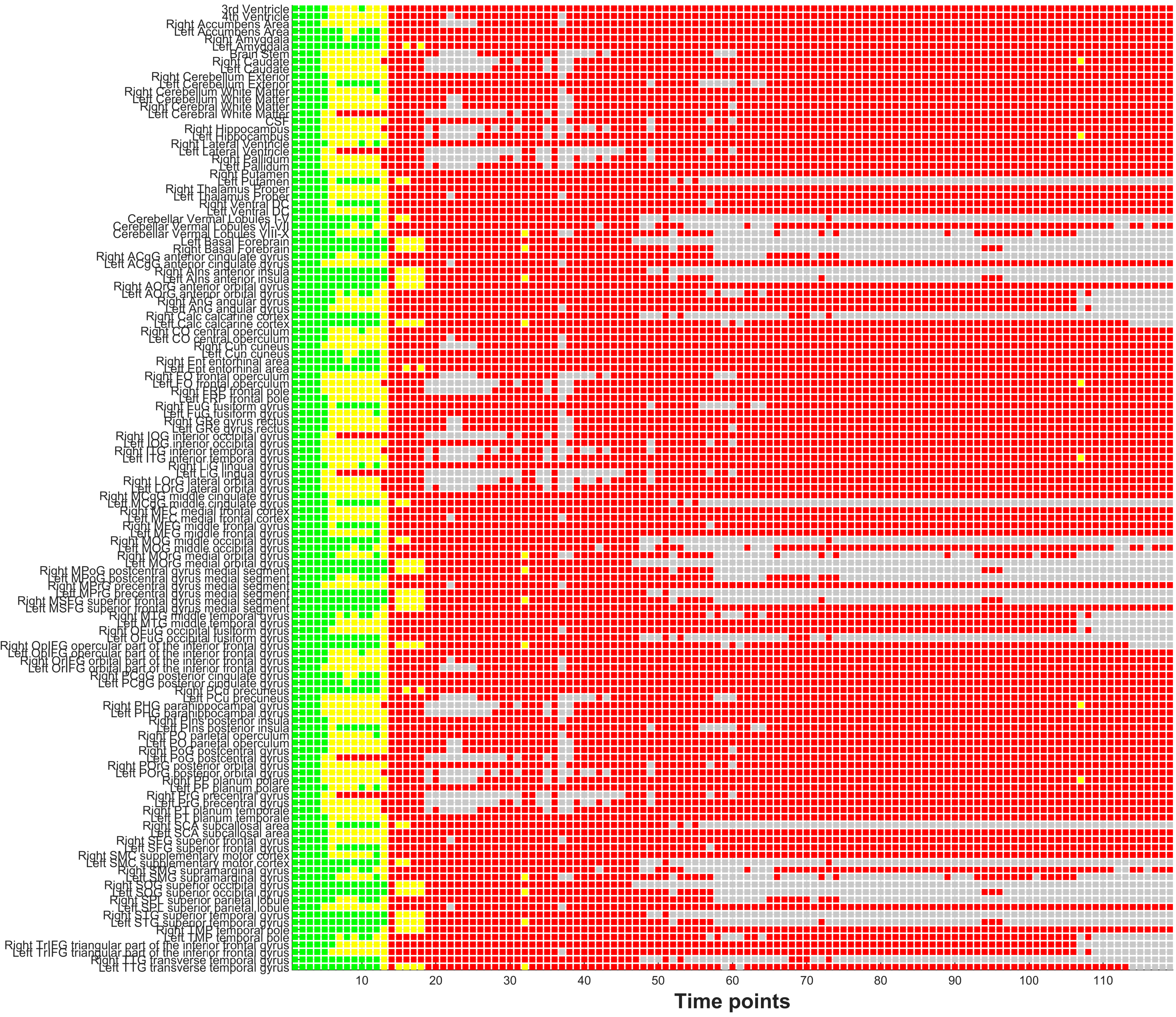

STS_OV in 129 regions of labels_Neuromorphometrics
(subject "typical"; > 90%: green; > 80%: yellow; > 55%: red; < 55%: gray)

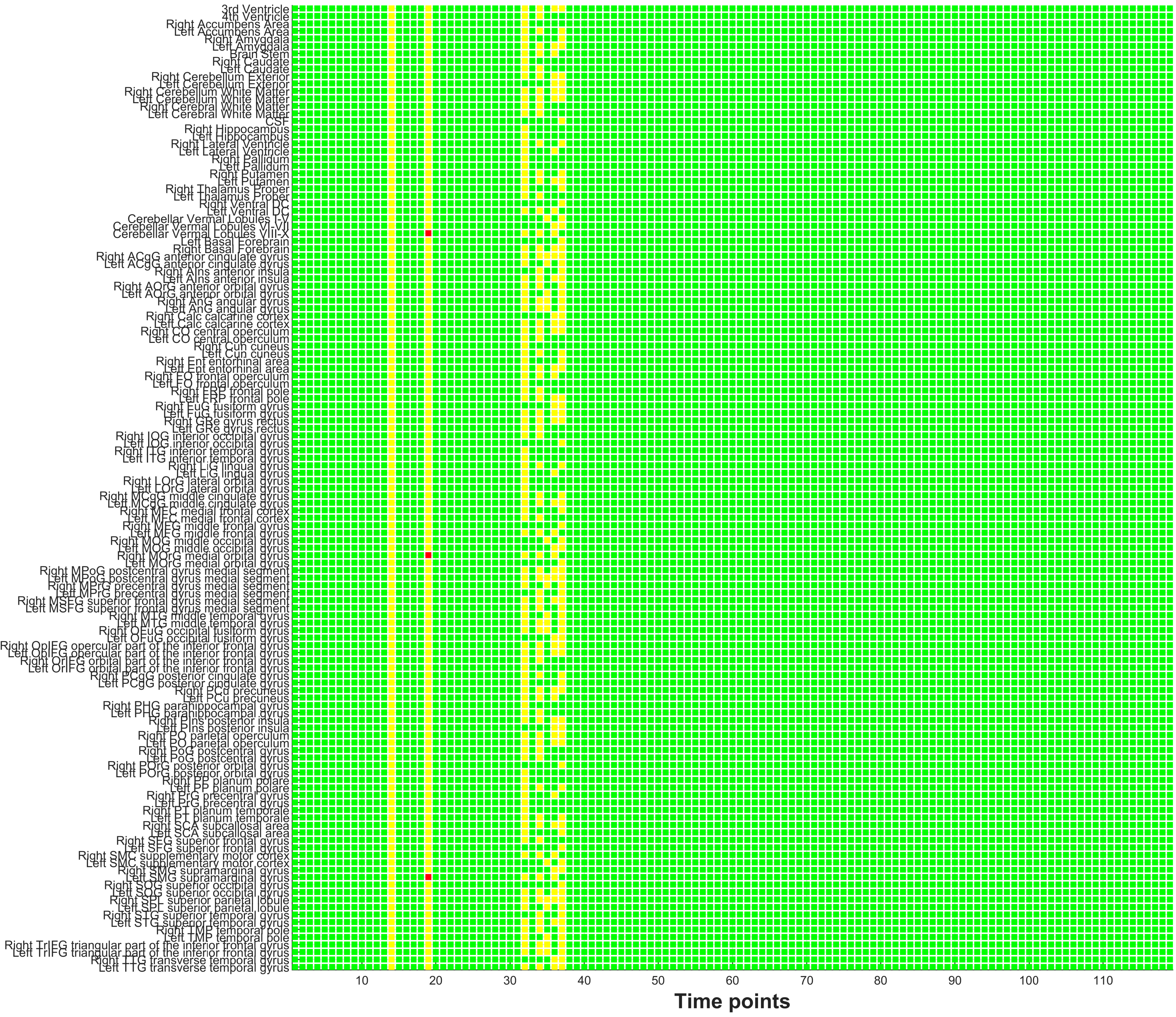

TD_OV in 127 regions of labels_Neuromorphometrics
(subject "bad"; > 90%: green; > 80%: yellow; > 55%: red; < 55%: gray)

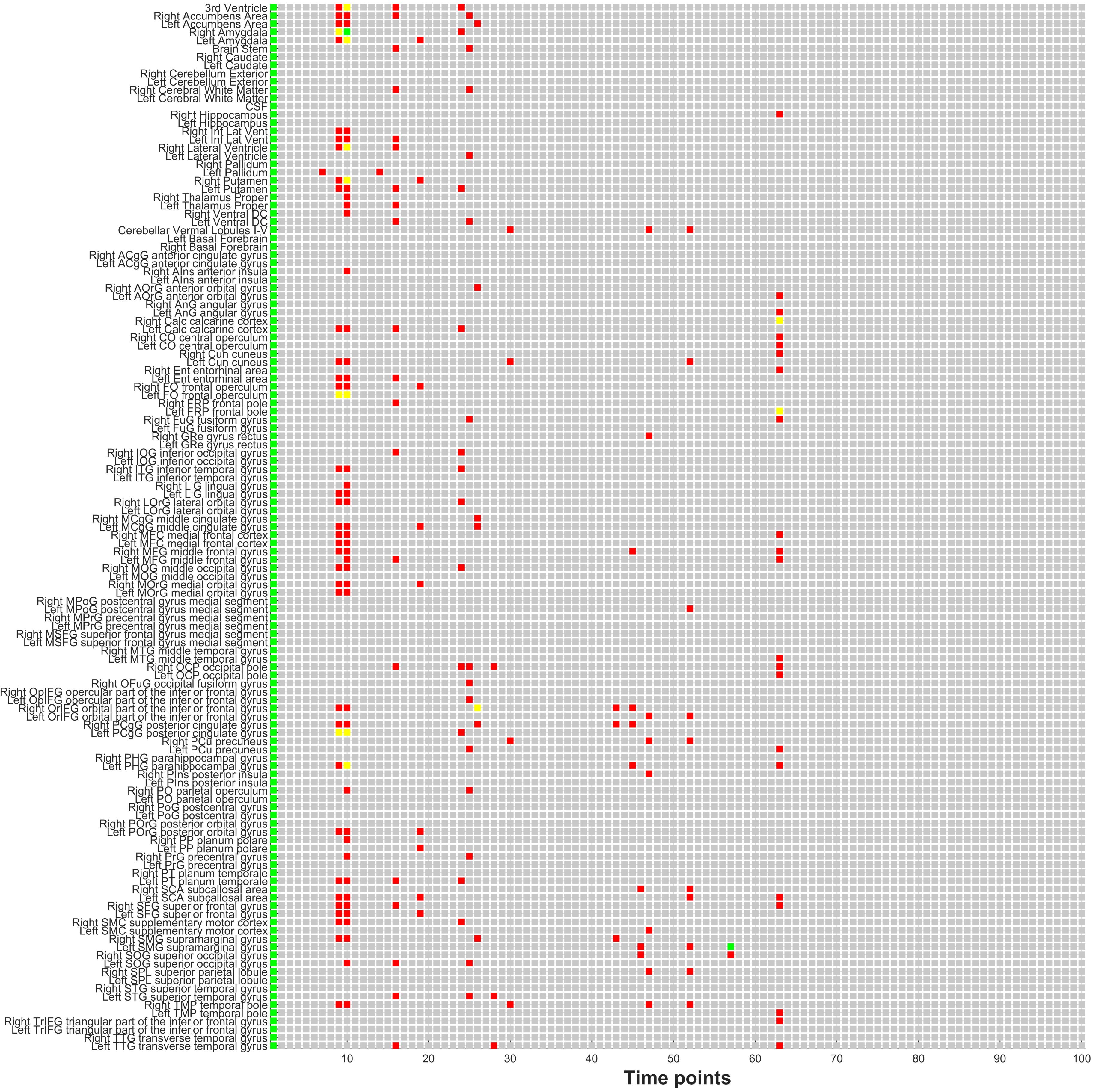

STS_OV in 127 regions of labels_Neuromorphometrics
(subject "bad"; > 90%: green; > 80%: yellow; > 55%: red; < 55%: gray)

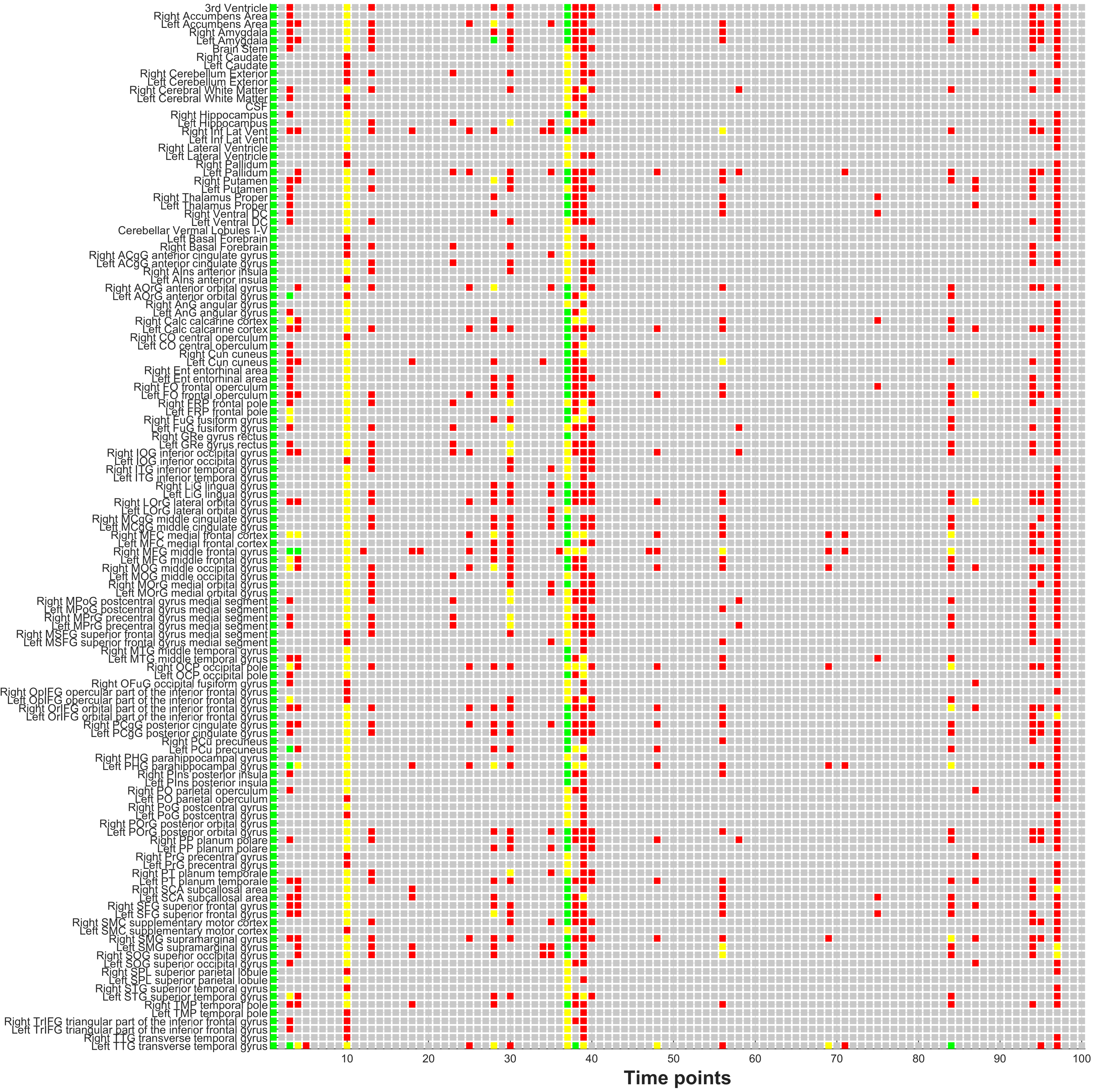

Supplement: Supplementary file 3 — Supplementary Figure 3 Representative examples of TDVVO and STSVVO for all regions in a standard neuroanatomical atlas (Neuromorphometrics atlas [www.neuromorphometrics.com] included with SPM), from the same “good”, “typical” or “bad” subjects shown in Supplementary Figure 2 and supplementary video material 1, 2, and 3. Note discrepancy between slow drifts (more notable in TDVVO) and fast motion (more notable in STSVVO). Also note spatial heterogeneity between regions (Y‐axis), again more obvious for TDVVO. [file HBM-46-e70337-s005.pdf]
