# Supplementary material for: Voxel Volume Overlap: Voxel‐Size Sensitive Indicators of Subject Motion in Functional MRI
Source: Hum Brain Mapp. 2025 Sep 9;46(13):e70337. doi: 10.1002/hbm.70337 (PMC12418571; doi:10.1002/hbm.70337)
Supplement: Supplementary file 7 — Supporting Information 4 Documentation of all individual subjects contained in datasets 1, 2, and 3. [file HBM-46-e70337-s004.pdf]

Supplementary material 1: details of all dataset

Dataset 1: high-quality resting state data, 712 sessions, 1 session per subject, 129.850 datapoints, from the fCONN1000 study; for more information, see http://fcon_1000.projects.nitrc.org/

Site	ID	Resolution in X [mm]	Resolution in Y [mm]	Resolution in Z [mm]	Sessions [n]	Datapoints [n]
Ann Arbor (b)	04111	3,44	3,44	3	1	295
Ann Arbor (b)	04619	3,44	3,44	3	1	295
Ann Arbor (b)	05580	3,44	3,44	3,20	1	395
Ann Arbor (b)	07921	3,44	3,44	3,20	1	395
Ann Arbor (b)	11043	3,44	3,44	3,20	1	395
Ann Arbor (b)	13636	3,44	3,44	3	1	295
Ann Arbor (b)	13959	3,44	3,44	3	1	295
Ann Arbor (b)	15846	3,44	3,44	3,20	1	395
Ann Arbor (b)	16960	3,44	3,44	3	1	295
Ann Arbor (b)	18546	3,44	3,44	3,20	1	395
Ann Arbor (b)	18698	3,44	3,44	3	1	295
Ann Arbor (b)	20317	3,44	3,44	3	1	295
Ann Arbor (b)	26099	3,44	3,44	3,20	1	395
Ann Arbor (b)	28433	3,44	3,44	3	1	295
Ann Arbor (b)	30250	3,44	3,44	3,20	1	395
Ann Arbor (b)	30421	3,44	3,44	3	1	295
Ann Arbor (b)	31218	3,44	3,44	3,20	1	395
Ann Arbor (b)	33437	3,44	3,44	3,20	1	395
Ann Arbor (b)	34781	3,44	3,44	3	1	295
Ann Arbor (b)	38614	3,44	3,44	3	1	295
Ann Arbor (b)	39635	3,44	3,44	3,20	1	395
Ann Arbor (b)	39923	3,44	3,44	3,20	1	395
Ann Arbor (b)	42616	3,44	3,44	3,20	1	395
Ann Arbor (b)	43409	3,44	3,44	3,20	1	395
Ann Arbor (b)	45569	3,44	3,44	3,20	1	395
Ann Arbor (b)	45660	3,44	3,44	3	1	295
Ann Arbor (b)	46727	3,44	3,44	3	1	295
Ann Arbor (b)	47659	3,44	3,44	3	1	295
Ann Arbor (b)	49687	3,44	3,44	3	1	295

Ann Arbor (b)	50859	3,44	3,44	3,20	1	395
Ann Arbor (b)	51248	3,44	3,44	3	1	295
Ann Arbor (b)	53269	3,44	3,44	3,20	1	395
Ann Arbor (b)	53959	3,44	3,44	3,20	1	395
Ann Arbor (b)	56028	3,44	3,44	3	1	295
Ann Arbor (b)	57025	3,44	3,44	3,20	1	395
Ann Arbor (b)	57196	3,44	3,44	3,20	1	395
Ann Arbor (b)	59573	3,44	3,44	3,20	1	395
Ann Arbor (b)	62819	3,44	3,44	3,20	1	395
Ann Arbor (b)	64831	3,44	3,44	3,20	1	395
Ann Arbor (b)	64969	3,44	3,44	3,20	1	395
Ann Arbor (b)	66506	3,44	3,44	3,20	1	395
Ann Arbor (b)	70106	3,44	3,44	3	1	295
Ann Arbor (b)	72215	3,44	3,44	3,20	1	395
Ann Arbor (b)	72416	3,44	3,44	3,20	1	395
Ann Arbor (b)	73168	3,44	3,44	3,20	1	395
Ann Arbor (b)	73812	3,44	3,44	3,20	1	395
Ann Arbor (b)	75922	3,44	3,44	3	1	295
Ann Arbor (b)	78151	3,44	3,44	3,20	1	395
Ann Arbor (b)	82334	3,44	3,44	3	1	295
Ann Arbor (b)	85257	3,44	3,44	3,20	1	395
Ann Arbor (b)	86367	3,44	3,44	3	1	295
Ann Arbor (b)	87745	3,44	3,44	3	1	295
Ann Arbor (b)	90127	3,44	3,44	3,20	1	395
Ann Arbor (b)	90950	3,44	3,44	3,20	1	395
Ann Arbor (b)	96621	3,44	3,44	3	1	295
Ann Arbor (b)	97518	3,44	3,44	3,20	1	395
Ann Arbor (b)	98007	3,44	3,44	3,20	1	395
Ann Arbor (b)	99692	3,44	3,44	3,20	1	395
Atlanta	00354	3,44	3,44	4	1	205
Atlanta	00368	3,44	3,44	4	1	205
Atlanta	06870	3,44	3,44	4	1	205
Atlanta	07145	3,44	3,44	4	1	205

Atlanta	15817	3,44	3,44	4	1	205
Atlanta	16563	3,44	3,44	4	1	205
Atlanta	18219	3,44	3,44	4	1	205
Atlanta	18702	3,44	3,44	4	1	205
Atlanta	24972	3,44	3,44	4	1	205
Atlanta	26938	3,44	3,44	4	1	205
Atlanta	32093	3,44	3,44	4	1	205
Atlanta	49816	3,44	3,44	4	1	205
Atlanta	52783	3,44	3,44	4	1	205
Atlanta	53122	3,44	3,44	4	1	205
Atlanta	55652	3,44	3,44	4	1	205
Atlanta	58250	3,44	3,44	4	1	205
Atlanta	59806	3,44	3,44	4	1	205
Atlanta	60499	3,44	3,44	4	1	205
Atlanta	61442	3,44	3,44	4	1	205
Atlanta	61902	3,44	3,44	4	1	205
Atlanta	71337	3,44	3,44	4	1	205
Atlanta	72096	3,44	3,44	4	1	205
Atlanta	72971	3,44	3,44	4	1	205
Atlanta	75153	3,44	3,44	4	1	205
Atlanta	76280	3,44	3,44	4	1	205
Atlanta	81596	3,44	3,44	4	1	205
Atlanta	86323	3,44	3,44	4	1	205
Atlanta	91049	3,44	3,44	4	1	205
Bangor	00031	3	3	3	1	265
Bangor	01903	3	3	3	1	265
Bangor	03557	3	3	3	1	265
Bangor	04097	3	3	3	1	265
Bangor	14388	3	3	3	1	265
Bangor	27519	3	3	3	1	265
Bangor	36736	3	3	3	1	265
Bangor	46870	3	3	3	1	265
Bangor	48632	3	3	3	1	265
Bangor	61418	3	3	3	1	265
Bangor	61908	3	3	3	1	265

Bangor	63767	3	3	3	1	265
Bangor	66585	3	3	3	1	265
Bangor	68050	3	3	3	1	265
Bangor	73082	3	3	3	1	265
Bangor	77520	3	3	3	1	265
Bangor	81464	3	3	3	1	265
Bangor	82625	3	3	3	1	265
Bangor	87568	3	3	3	1	265
Bangor	91556	3	3	3	1	265
Beijing (Zang)	00440	3,13	3,13	3,60	1	225
Beijing (Zang)	01018	3,13	3,13	3,60	1	225
Beijing (Zang)	01244	3,13	3,13	3,60	1	225
Beijing (Zang)	02403	3,13	3,13	3,60	1	225
Beijing (Zang)	04050	3,13	3,13	3,60	1	225
Beijing (Zang)	04191	3,13	3,13	3,60	1	225
Beijing (Zang)	05267	3,13	3,13	3,60	1	225
Beijing (Zang)	06880	3,13	3,13	3,60	1	225
Beijing (Zang)	06899	3,13	3,13	3,60	1	225
Beijing (Zang)	07144	3,13	3,13	3,60	1	225
Beijing (Zang)	07716	3,13	3,13	3,60	1	225
Beijing (Zang)	07717	3,13	3,13	3,60	1	225
Beijing (Zang)	08001	3,13	3,13	3,60	1	225
Beijing (Zang)	08251	3,13	3,13	3,60	1	225
Beijing (Zang)	08455	3,13	3,13	3,60	1	225
Beijing (Zang)	08816	3,13	3,13	3,60	1	225
Beijing (Zang)	08992	3,13	3,13	3,60	1	225
Beijing (Zang)	10186	3,13	3,13	3,60	1	225
Beijing (Zang)	10277	3,13	3,13	3,60	1	225
Beijing (Zang)	10869	3,13	3,13	3,60	1	225
Beijing (Zang)	10973	3,13	3,13	3,60	1	225
Beijing (Zang)	11072	3,13	3,13	3,60	1	225
Beijing (Zang)	11344	3,13	3,13	3,60	1	225
Beijing (Zang)	12220	3,13	3,13	3,60	1	225
Beijing (Zang)	14238	3,13	3,13	3,60	1	225

Beijing (Zang)	15441	3,13	3,13	3,60	1	225
Beijing (Zang)	16091	3,13	3,13	3,60	1	225
Beijing (Zang)	16943	3,13	3,13	3,60	1	225
Beijing (Zang)	17093	3,13	3,13	3,60	1	225
Beijing (Zang)	17159	3,13	3,13	3,60	1	225
Beijing (Zang)	17315	3,13	3,13	3,60	1	225
Beijing (Zang)	17586	3,13	3,13	3,60	1	225
Beijing (Zang)	17603	3,13	3,13	3,60	1	225
Beijing (Zang)	17642	3,13	3,13	3,60	1	225
Beijing (Zang)	17688	3,13	3,13	3,60	1	225
Beijing (Zang)	18326	3,13	3,13	3,60	1	225
Beijing (Zang)	18758	3,13	3,13	3,60	1	225
Beijing (Zang)	18960	3,13	3,13	3,60	1	225
Beijing (Zang)	19642	3,13	3,13	3,60	1	225
Beijing (Zang)	19974	3,13	3,13	3,60	1	225
Beijing (Zang)	20127	3,13	3,13	3,60	1	225
Beijing (Zang)	20246	3,13	3,13	3,60	1	225
Beijing (Zang)	20765	3,13	3,13	3,60	1	225
Beijing (Zang)	20948	3,13	3,13	3,60	1	225
Beijing (Zang)	21115	3,13	3,13	3,60	1	225
Beijing (Zang)	22201	3,13	3,13	3,60	1	225
Beijing (Zang)	22595	3,13	3,13	3,60	1	225
Beijing (Zang)	22661	3,13	3,13	3,60	1	225
Beijing (Zang)	22715	3,13	3,13	3,60	1	225
Beijing (Zang)	22890	3,13	3,13	3,60	1	225
Beijing (Zang)	26713	3,13	3,13	3,60	1	225
Beijing (Zang)	28206	3,13	3,13	3,60	1	225
Beijing (Zang)	28403	3,13	3,13	3,60	1	225
Beijing (Zang)	28698	3,13	3,13	3,60	1	225
Beijing (Zang)	28792	3,13	3,13	3,60	1	225
Beijing (Zang)	28801	3,13	3,13	3,60	1	225
Beijing (Zang)	28907	3,13	3,13	3,60	1	225
Beijing (Zang)	28965	3,13	3,13	3,60	1	225

Beijing (Zang)	29590	3,13	3,13	3,60	1	225
Beijing (Zang)	29785	3,13	3,13	3,60	1	225
Beijing (Zang)	30272	3,13	3,13	3,60	1	225
Beijing (Zang)	30310	3,13	3,13	3,60	1	225
Beijing (Zang)	30556	3,13	3,13	3,60	1	225
Beijing (Zang)	30616	3,13	3,13	3,60	1	225
Beijing (Zang)	30988	3,13	3,13	3,60	1	225
Beijing (Zang)	31058	3,13	3,13	3,60	1	225
Beijing (Zang)	31729	3,13	3,13	3,60	1	225
Beijing (Zang)	32517	3,13	3,13	3,60	1	225
Beijing (Zang)	32587	3,13	3,13	3,60	1	225
Beijing (Zang)	33747	3,13	3,13	3,60	1	225
Beijing (Zang)	33943	3,13	3,13	3,60	1	225
Beijing (Zang)	33991	3,13	3,13	3,60	1	225
Beijing (Zang)	34895	3,13	3,13	3,60	1	225
Beijing (Zang)	34943	3,13	3,13	3,60	1	225
Beijing (Zang)	35309	3,13	3,13	3,60	1	225
Beijing (Zang)	35776	3,13	3,13	3,60	1	225
Beijing (Zang)	35806	3,13	3,13	3,60	1	225
Beijing (Zang)	36580	3,13	3,13	3,60	1	225
Beijing (Zang)	36942	3,13	3,13	3,60	1	225
Beijing (Zang)	37602	3,13	3,13	3,60	1	225
Beijing (Zang)	38602	3,13	3,13	3,60	1	225
Beijing (Zang)	39725	3,13	3,13	3,60	1	225
Beijing (Zang)	40037	3,13	3,13	3,60	1	225
Beijing (Zang)	40427	3,13	3,13	3,60	1	225
Beijing (Zang)	41170	3,13	3,13	3,60	1	225
Beijing (Zang)	41621	3,13	3,13	3,60	1	225
Beijing (Zang)	42512	3,13	3,13	3,60	1	225
Beijing (Zang)	42555	3,13	3,13	3,60	1	225
Beijing (Zang)	42843	3,13	3,13	3,60	1	225
Beijing (Zang)	43290	3,13	3,13	3,60	1	225
Beijing (Zang)	44573	3,13	3,13	3,60	1	225

Beijing (Zang)	45552	3,13	3,13	3,60	1	225
Beijing (Zang)	46058	3,13	3,13	3,60	1	225
Beijing (Zang)	46259	3,13	3,13	3,60	1	225
Beijing (Zang)	46541	3,13	3,13	3,60	1	225
Beijing (Zang)	48501	3,13	3,13	3,60	1	225
Beijing (Zang)	48563	3,13	3,13	3,60	1	225
Beijing (Zang)	48676	3,13	3,13	3,60	1	225
Beijing (Zang)	49782	3,13	3,13	3,60	1	225
Beijing (Zang)	50498	3,13	3,13	3,60	1	225
Beijing (Zang)	50873	3,13	3,13	3,60	1	225
Beijing (Zang)	50972	3,13	3,13	3,60	1	225
Beijing (Zang)	50985	3,13	3,13	3,60	1	225
Beijing (Zang)	51015	3,13	3,13	3,60	1	225
Beijing (Zang)	51586	3,13	3,13	3,60	1	225
Beijing (Zang)	52044	3,13	3,13	3,60	1	225
Beijing (Zang)	52259	3,13	3,13	3,60	1	225
Beijing (Zang)	53572	3,13	3,13	3,60	1	225
Beijing (Zang)	53998	3,13	3,13	3,60	1	225
Beijing (Zang)	54890	3,13	3,13	3,60	1	225
Beijing (Zang)	55301	3,13	3,13	3,60	1	225
Beijing (Zang)	55541	3,13	3,13	3,60	1	225
Beijing (Zang)	55736	3,13	3,13	3,60	1	225
Beijing (Zang)	55856	3,13	3,13	3,60	1	225
Beijing (Zang)	56136	3,13	3,13	3,60	1	225
Beijing (Zang)	56659	3,13	3,13	3,60	1	225
Beijing (Zang)	56703	3,13	3,13	3,60	1	225
Beijing (Zang)	56757	3,13	3,13	3,60	1	225
Beijing (Zang)	58029	3,13	3,13	3,60	1	225
Beijing (Zang)	58332	3,13	3,13	3,60	1	225
Beijing (Zang)	58614	3,13	3,13	3,60	1	225
Beijing (Zang)	59347	3,13	3,13	3,60	1	225
Beijing (Zang)	59448	3,13	3,13	3,60	1	225
Beijing (Zang)	61961	3,13	3,13	3,60	1	225

Beijing (Zang)	62083	3,13	3,13	3,60	1	225
Beijing (Zang)	62438	3,13	3,13	3,60	1	225
Beijing (Zang)	62843	3,13	3,13	3,60	1	225
Beijing (Zang)	62966	3,60	3,13	3,13	1	225
Beijing (Zang)	64923	3,13	3,13	3,60	1	225
Beijing (Zang)	65467	3,13	3,13	3,60	1	225
Beijing (Zang)	65659	3,13	3,13	3,60	1	225
Beijing (Zang)	66158	3,13	3,13	3,60	1	225
Beijing (Zang)	66528	3,13	3,13	3,60	1	225
Beijing (Zang)	66781	3,13	3,13	3,60	1	225
Beijing (Zang)	66889	3,13	3,13	3,60	1	225
Beijing (Zang)	67435	3,13	3,13	3,60	1	225
Beijing (Zang)	67844	3,13	3,13	3,60	1	225
Beijing (Zang)	68012	3,13	3,13	3,60	1	225
Beijing (Zang)	68597	3,13	3,13	3,60	1	225
Beijing (Zang)	69518	3,13	3,13	3,60	1	225
Beijing (Zang)	69696	3,13	3,13	3,60	1	225
Beijing (Zang)	71693	3,13	3,13	3,60	1	225
Beijing (Zang)	72654	3,13	3,13	3,60	1	225
Beijing (Zang)	72678	3,13	3,13	3,60	1	225
Beijing (Zang)	73098	3,13	3,13	3,60	1	225
Beijing (Zang)	73245	3,13	3,13	3,60	1	225
Beijing (Zang)	73279	3,13	3,13	3,60	1	225
Beijing (Zang)	73421	3,13	3,13	3,60	1	225
Beijing (Zang)	74386	3,13	3,13	3,60	1	225
Beijing (Zang)	74587	3,13	3,13	3,60	1	225
Beijing (Zang)	75878	3,13	3,13	3,60	1	225
Beijing (Zang)	76377	3,13	3,13	3,60	1	225
Beijing (Zang)	77440	3,13	3,13	3,60	1	225
Beijing (Zang)	80163	3,13	3,13	3,60	1	225
Beijing (Zang)	80551	3,13	3,13	3,60	1	225
Beijing (Zang)	80569	3,13	3,13	3,60	1	225
Beijing (Zang)	80927	3,13	3,13	3,60	1	225

Beijing (Zang)	81062	3,13	3,13	3,60	1	225
Beijing (Zang)	81074	3,13	3,13	3,60	1	225
Beijing (Zang)	82352	3,13	3,13	3,60	1	225
Beijing (Zang)	82426	3,13	3,13	3,60	1	225
Beijing (Zang)	82714	3,13	3,13	3,60	1	225
Beijing (Zang)	82826	3,13	3,13	3,60	1	225
Beijing (Zang)	82980	3,13	3,13	3,60	1	225
Beijing (Zang)	83430	3,13	3,13	3,60	1	225
Beijing (Zang)	83624	3,13	3,13	3,60	1	225
Beijing (Zang)	83728	3,13	3,13	3,60	1	225
Beijing (Zang)	85030	3,13	3,13	3,60	1	225
Beijing (Zang)	85543	3,13	3,13	3,60	1	225
Beijing (Zang)	85818	3,13	3,13	3,60	1	225
Beijing (Zang)	86114	3,13	3,13	3,60	1	225
Beijing (Zang)	87089	3,13	3,13	3,60	1	225
Beijing (Zang)	87776	3,13	3,13	3,60	1	225
Beijing (Zang)	88306	3,13	3,13	3,60	1	225
Beijing (Zang)	88947	3,13	3,13	3,60	1	225
Beijing (Zang)	89088	3,13	3,13	3,60	1	225
Beijing (Zang)	89238	3,13	3,13	3,60	1	225
Beijing (Zang)	89592	3,13	3,13	3,60	1	225
Beijing (Zang)	89742	3,13	3,13	3,60	1	225
Beijing (Zang)	89941	3,13	3,13	3,60	1	225
Beijing (Zang)	91145	3,13	3,13	3,60	1	225
Beijing (Zang)	91399	3,13	3,13	3,60	1	225
Beijing (Zang)	91952	3,13	3,13	3,60	1	225
Beijing (Zang)	92430	3,13	3,13	3,60	1	225
Beijing (Zang)	92490	3,13	3,13	3,60	1	225
Beijing (Zang)	92544	3,13	3,13	3,60	1	225
Beijing (Zang)	92602	3,13	3,13	3,60	1	225
Beijing (Zang)	92799	3,13	3,13	3,60	1	225
Beijing (Zang)	92859	3,13	3,13	3,60	1	225
Beijing (Zang)	93689	3,13	3,13	3,60	1	225

Beijing (Zang)	93856	3,13	3,13	3,60	1	225
Beijing (Zang)	94536	3,13	3,13	3,60	1	225
Beijing (Zang)	95575	3,13	3,13	3,60	1	225
Beijing (Zang)	95755	3,13	3,13	3,60	1	225
Beijing (Zang)	96163	3,13	3,13	3,60	1	225
Beijing (Zang)	97442	3,13	3,13	3,60	1	225
Beijing (Zang)	98353	3,13	3,13	3,60	1	225
Beijing (Zang)	98617	3,13	3,13	3,60	1	225
Berlin (Schmidt)	17017	2,67	2,67	3	1	123
Berlin (Schmidt)	23927	2,67	2,67	3	1	123
Berlin (Schmidt)	29158	2,67	2,67	3	1	123
Berlin (Schmidt)	30072	2,67	2,67	3	1	123
Berlin (Schmidt)	31837	2,67	2,67	3	1	123
Berlin (Schmidt)	37548	2,67	2,67	3	1	123
Berlin (Schmidt)	52358	2,67	2,67	3	1	123
Berlin (Schmidt)	54257	2,67	2,67	3	1	123
Berlin (Schmidt)	54329	2,67	2,67	3	1	123
Berlin (Schmidt)	73823	2,67	2,67	3	1	123
Berlin (Schmidt)	76160	2,67	2,67	3	1	123
Berlin (Schmidt)	77572	2,67	2,67	3	1	123
Berlin (Schmidt)	80221	2,67	2,67	3	1	123
Berlin (Schmidt)	81887	2,67	2,67	3	1	123
Berlin (Schmidt)	85922	2,67	2,67	3	1	123
Berlin (Schmidt)	86414	2,67	2,67	3	1	123
Berlin (Schmidt)	90658	2,67	2,67	3	1	123
Berlin (Schmidt)	90893	2,67	2,67	3	1	123
Berlin (Schmidt)	91622	2,67	2,67	3	1	123
Berlin (Schmidt)	94042	2,67	2,67	3	1	123
Berlin (Schmidt)	96234	2,67	2,67	3	1	123
Berlin (Margulies)	06204	3	3	4	1	195
Berlin (Margulies)	06716	3	3	4	1	195
Berlin (Margulies)	12855	3	3	4	1	195
Berlin (Margulies)	18913	3	3	4	1	195

Berlin (Margulies)	23506	3	3	4	1	195
Berlin (Margulies)	27536	3	3	4	1	195
Berlin (Margulies)	27711	3	3	4	1	195
Berlin (Margulies)	27797	3	3	4	1	195
Berlin (Margulies)	28092	3	3	4	1	195
Berlin (Margulies)	33248	3	3	4	1	195
Berlin (Margulies)	38279	3	3	4	1	195
Berlin (Margulies)	40143	3	3	4	1	195
Berlin (Margulies)	47066	3	3	4	1	195
Berlin (Margulies)	47791	3	3	4	1	195
Berlin (Margulies)	49134	3	3	4	1	195
Berlin (Margulies)	54976	3	3	4	1	195
Berlin (Margulies)	57028	3	3	4	1	195
Berlin (Margulies)	67166	3	3	4	1	195
Berlin (Margulies)	75506	3	3	4	1	195
Berlin (Margulies)	77281	3	3	4	1	195
Berlin (Margulies)	85681	3	3	4	1	195
Berlin (Margulies)	86111	3	3	4	1	195
Berlin (Margulies)	91116	3	3	4	1	195
Berlin (Margulies)	91966	3	3	4	1	195
Berlin (Margulies)	95068	3	3	4	1	195
Berlin (Margulies)	97162	3	3	4	1	195
Cambridge (Buckner)	00156	3	3	3	1	119
Cambridge (Buckner)	00294	3	3	3	1	119
Cambridge (Buckner)	01361	3	3	3	1	119
Cambridge (Buckner)	02591	3	3	3	1	119
Cambridge (Buckner)	02953	3	3	3	1	119
Cambridge (Buckner)	04187	3	3	3	1	119
Cambridge (Buckner)	04270	3	3	3	1	119
Cambridge (Buckner)	04491	3	3	3	1	119
Cambridge (Buckner)	04665	3	3	3	1	119
Cambridge (Buckner)	05306	3	3	3	1	119
Cambridge (Buckner)	05453	3	3	3	1	119

Cambridge (Buckner)	06037	3	3	3	1	119
Cambridge (Buckner)	06272	3	3	3	1	119
Cambridge (Buckner)	06987	3	3	3	1	119
Cambridge (Buckner)	07413	3	3	3	1	119
Cambridge (Buckner)	07798	3	3	3	1	119
Cambridge (Buckner)	07902	3	3	3	1	119
Cambridge (Buckner)	08204	3	3	3	1	119
Cambridge (Buckner)	08588	3	3	3	1	119
Cambridge (Buckner)	08723	3	3	3	1	119
Cambridge (Buckner)	08947	3	3	3	1	119
Cambridge (Buckner)	09015	3	3	3	1	119
Cambridge (Buckner)	09397	3	3	3	1	119
Cambridge (Buckner)	09633	3	3	3	1	119
Cambridge (Buckner)	10268	3	3	3	1	119
Cambridge (Buckner)	10619	3	3	3	1	119
Cambridge (Buckner)	11388	3	3	3	1	119
Cambridge (Buckner)	12346	3	3	3	1	119
Cambridge (Buckner)	13093	3	3	3	1	119
Cambridge (Buckner)	13187	3	3	3	1	119
Cambridge (Buckner)	13216	3	3	3	1	119
Cambridge (Buckner)	13902	3	3	3	1	119
Cambridge (Buckner)	14183	3	3	3	1	119
Cambridge (Buckner)	14194	3	3	3	1	119
Cambridge (Buckner)	14278	3	3	3	1	119
Cambridge (Buckner)	15172	3	3	3	1	119
Cambridge (Buckner)	15258	3	3	3	1	119
Cambridge (Buckner)	15432	3	3	3	1	119
Cambridge (Buckner)	15905	3	3	3	1	119
Cambridge (Buckner)	16122	3	3	3	1	119
Cambridge (Buckner)	16390	3	3	3	1	119
Cambridge (Buckner)	16846	3	3	3	1	119
Cambridge (Buckner)	17584	3	3	3	1	119
Cambridge (Buckner)	17737	3	3	3	1	119

Cambridge (Buckner)	17772	3	3	3	1	119
Cambridge (Buckner)	18295	3	3	3	1	119
Cambridge (Buckner)	18449	3	3	3	1	119
Cambridge (Buckner)	19717	3	3	3	1	119
Cambridge (Buckner)	20389	3	3	3	1	119
Cambridge (Buckner)	20543	3	3	3	1	119
Cambridge (Buckner)	20563	3	3	3	1	119
Cambridge (Buckner)	21755	3	3	3	1	119
Cambridge (Buckner)	23780	3	3	3	1	119
Cambridge (Buckner)	23869	3	3	3	1	119
Cambridge (Buckner)	24670	3	3	3	1	119
Cambridge (Buckner)	24757	3	3	3	1	119
Cambridge (Buckner)	25044	3	3	3	1	119
Cambridge (Buckner)	25058	3	3	3	1	119
Cambridge (Buckner)	25099	3	3	3	1	119
Cambridge (Buckner)	26348	3	3	3	1	119
Cambridge (Buckner)	27065	3	3	3	1	119
Cambridge (Buckner)	27230	3	3	3	1	119
Cambridge (Buckner)	27613	3	3	3	1	119
Cambridge (Buckner)	27796	3	3	3	1	119
Cambridge (Buckner)	29044	3	3	3	1	119
Cambridge (Buckner)	29425	3	3	3	1	119
Cambridge (Buckner)	29800	3	3	3	1	119
Cambridge (Buckner)	31522	3	3	3	1	119
Cambridge (Buckner)	34586	3	3	3	1	119
Cambridge (Buckner)	34741	3	3	3	1	119
Cambridge (Buckner)	35430	3	3	3	1	119
Cambridge (Buckner)	35512	3	3	3	1	119
Cambridge (Buckner)	37374	3	3	3	1	119
Cambridge (Buckner)	39053	3	3	3	1	119
Cambridge (Buckner)	39065	3	3	3	1	119
Cambridge (Buckner)	39142	3	3	3	1	119
Cambridge (Buckner)	39737	3	3	3	1	119

Cambridge (Buckner)	40635	3	3	3	1	119
Cambridge (Buckner)	41567	3	3	3	1	119
Cambridge (Buckner)	41773	3	3	3	1	119
Cambridge (Buckner)	41814	3	3	3	1	119
Cambridge (Buckner)	42146	3	3	3	1	119
Cambridge (Buckner)	42253	3	3	3	1	119
Cambridge (Buckner)	43304	3	3	3	1	119
Cambridge (Buckner)	43358	3	3	3	1	119
Cambridge (Buckner)	45344	3	3	3	1	119
Cambridge (Buckner)	45354	3	3	3	1	119
Cambridge (Buckner)	45604	3	3	3	1	119
Cambridge (Buckner)	47162	3	3	3	1	119
Cambridge (Buckner)	47231	3	3	3	1	119
Cambridge (Buckner)	47278	3	3	3	1	119
Cambridge (Buckner)	47498	3	3	3	1	119
Cambridge (Buckner)	49259	3	3	3	1	119
Cambridge (Buckner)	49998	3	3	3	1	119
Cambridge (Buckner)	50272	3	3	3	1	119
Cambridge (Buckner)	50454	3	3	3	1	119
Cambridge (Buckner)	50953	3	3	3	1	119
Cambridge (Buckner)	51050	3	3	3	1	119
Cambridge (Buckner)	51172	3	3	3	1	119
Cambridge (Buckner)	51512	3	3	3	1	119
Cambridge (Buckner)	51671	3	3	3	1	119
Cambridge (Buckner)	52036	3	3	3	1	119
Cambridge (Buckner)	52300	3	3	3	1	119
Cambridge (Buckner)	52442	3	3	3	1	119
Cambridge (Buckner)	53059	3	3	3	1	119
Cambridge (Buckner)	53107	3	3	3	1	119
Cambridge (Buckner)	53193	3	3	3	1	119
Cambridge (Buckner)	53296	3	3	3	1	119
Cambridge (Buckner)	53615	3	3	3	1	119
Cambridge (Buckner)	54846	3	3	3	1	119

Cambridge (Buckner)	55114	3	3	3	1	119
Cambridge (Buckner)	55660	3	3	3	1	119
Cambridge (Buckner)	55874	3	3	3	1	119
Cambridge (Buckner)	57221	3	3	3	1	119
Cambridge (Buckner)	58360	3	3	3	1	119
Cambridge (Buckner)	58470	3	3	3	1	119
Cambridge (Buckner)	58682	3	3	3	1	119
Cambridge (Buckner)	58874	3	3	3	1	119
Cambridge (Buckner)	59434	3	3	3	1	119
Cambridge (Buckner)	59729	3	3	3	1	119
Cambridge (Buckner)	60578	3	3	3	1	119
Cambridge (Buckner)	60797	3	3	3	1	119
Cambridge (Buckner)	61185	3	3	3	1	119
Cambridge (Buckner)	61209	3	3	3	1	119
Cambridge (Buckner)	61436	3	3	3	1	119
Cambridge (Buckner)	61753	3	3	3	1	119
Cambridge (Buckner)	62383	3	3	3	1	119
Cambridge (Buckner)	62424	3	3	3	1	119
Cambridge (Buckner)	62732	3	3	3	1	119
Cambridge (Buckner)	62908	3	3	3	1	119
Cambridge (Buckner)	63412	3	3	3	1	119
Cambridge (Buckner)	63661	3	3	3	1	119
Cambridge (Buckner)	64308	3	3	3	1	119
Cambridge (Buckner)	64985	3	3	3	1	119
Cambridge (Buckner)	65373	3	3	3	1	119
Cambridge (Buckner)	65682	3	3	3	1	119
Cambridge (Buckner)	66351	3	3	3	1	119
Cambridge (Buckner)	67117	3	3	3	1	119
Cambridge (Buckner)	68101	3	3	3	1	119
Cambridge (Buckner)	68425	3	3	3	1	119
Cambridge (Buckner)	69287	3	3	3	1	119
Cambridge (Buckner)	69315	3	3	3	1	119
Cambridge (Buckner)	69397	3	3	3	1	119

Cambridge (Buckner)	71849	3	3	3	1	119
Cambridge (Buckner)	72068	3	3	3	1	119
Cambridge (Buckner)	73317	3	3	3	1	119
Cambridge (Buckner)	73399	3	3	3	1	119
Cambridge (Buckner)	73477	3	3	3	1	119
Cambridge (Buckner)	76631	3	3	3	1	119
Cambridge (Buckner)	76745	3	3	3	1	119
Cambridge (Buckner)	77337	3	3	3	1	119
Cambridge (Buckner)	77435	3	3	3	1	119
Cambridge (Buckner)	77598	3	3	3	1	119
Cambridge (Buckner)	77989	3	3	3	1	119
Cambridge (Buckner)	78547	3	3	3	1	119
Cambridge (Buckner)	78552	3	3	3	1	119
Cambridge (Buckner)	78614	3	3	3	1	119
Cambridge (Buckner)	80557	3	3	3	1	119
Cambridge (Buckner)	81289	3	3	3	1	119
Cambridge (Buckner)	81524	3	3	3	1	119
Cambridge (Buckner)	81562	3	3	3	1	119
Cambridge (Buckner)	82113	3	3	3	1	119
Cambridge (Buckner)	82213	3	3	3	1	119
Cambridge (Buckner)	82435	3	3	3	1	119
Cambridge (Buckner)	83409	3	3	3	1	119
Cambridge (Buckner)	83683	3	3	3	1	119
Cambridge (Buckner)	84064	3	3	3	1	119
Cambridge (Buckner)	84256	3	3	3	1	119
Cambridge (Buckner)	84504	3	3	3	1	119
Cambridge (Buckner)	84845	3	3	3	1	119
Cambridge (Buckner)	86115	3	3	3	1	119
Cambridge (Buckner)	86637	3	3	3	1	119
Cambridge (Buckner)	87846	3	3	3	1	119
Cambridge (Buckner)	88445	3	3	3	1	119
Cambridge (Buckner)	88464	3	3	3	1	119
Cambridge (Buckner)	88853	3	3	3	1	119

Cambridge (Buckner)	89107	3	3	3	1	119
Cambridge (Buckner)	89435	3	3	3	1	119
Cambridge (Buckner)	89894	3	3	3	1	119
Cambridge (Buckner)	90059	3	3	3	1	119
Cambridge (Buckner)	90674	3	3	3	1	119
Cambridge (Buckner)	90681	3	3	3	1	119
Cambridge (Buckner)	90699	3	3	3	1	119
Cambridge (Buckner)	92288	3	3	3	1	119
Cambridge (Buckner)	92393	3	3	3	1	119
Cambridge (Buckner)	92440	3	3	3	1	119
Cambridge (Buckner)	93269	3	3	3	1	119
Cambridge (Buckner)	93488	3	3	3	1	119
Cambridge (Buckner)	93609	3	3	3	1	119
Cambridge (Buckner)	94304	3	3	3	1	119
Cambridge (Buckner)	95187	3	3	3	1	119
Cambridge (Buckner)	95644	3	3	3	1	119
Cambridge (Buckner)	95959	3	3	3	1	119
Cambridge (Buckner)	98528	3	3	3	1	119
Cambridge (Buckner)	98624	3	3	3	1	119
Cambridge (Buckner)	99085	3	3	3	1	119
Cambridge (Buckner)	99330	3	3	3	1	119
Cambridge (Buckner)	99462	3	3	3	1	119
Cleveland	02480	2	2	4	1	127
Cleveland	07835	2	2	4	1	127
Cleveland	12330	2	2	4	1	127
Cleveland	13495	2	2	4	1	127
Cleveland	17946	2	2	4	1	127
Cleveland	18011	2	2	4	1	127
Cleveland	18566	2	2	4	1	127
Cleveland	19005	2	2	4	1	127
Cleveland	20003	2	2	4	1	127
Cleveland	22736	2	2	4	1	127
Cleveland	22935	2	2	4	1	127
Cleveland	26557	2	2	4	1	127

Cleveland	28596	2	2	4	1	127
Cleveland	34189	2	2	4	1	127
Cleveland	46075	2	2	4	1	127
Cleveland	46739	2	2	4	1	127
Cleveland	47482	2	2	4	1	127
Cleveland	50092	2	2	4	1	127
Cleveland	58811	2	2	4	1	127
Cleveland	61868	2	2	4	1	127
Cleveland	64706	2	2	4	1	127
Cleveland	65858	2	2	4	1	127
Cleveland	67936	2	2	4	1	127
Cleveland	75398	2	2	4	1	127
Cleveland	76139	2	2	4	1	127
Cleveland	80263	2	2	4	1	127
Cleveland	82518	2	2	4	1	127
Cleveland	85091	2	2	4	1	127
Cleveland	92232	2	2	4	1	127
Cleveland	97844	2	2	4	1	127
Cleveland	99664	2	2	4	1	127
Dallas	04288	3,44	3,44	4	1	115
Dallas	05892	3,44	3,44	4	1	115
Dallas	16493	3,44	3,44	4	1	115
Dallas	19940	3,44	3,44	4	1	115
Dallas	25085	3,44	3,44	4	1	115
Dallas	32183	3,44	3,44	4	1	115
Dallas	32272	3,44	3,44	4	1	115
Dallas	40897	3,44	3,44	4	1	115
Dallas	45978	3,44	3,44	4	1	115
Dallas	51824	3,44	3,44	4	1	115
Dallas	57450	3,44	3,44	4	1	115
Dallas	58347	3,44	3,44	4	1	115
Dallas	58803	3,44	3,44	4	1	115
Dallas	59610	3,44	3,44	4	1	115
Dallas	71043	3,44	3,44	4	1	115
Dallas	77150	3,44	3,44	4	1	115

Dallas	79426	3,44	3,44	4	1	115
Dallas	80418	3,44	3,44	4	1	115
Dallas	81423	3,44	3,44	4	1	115
Dallas	83998	3,44	3,44	4	1	115
Dallas	85470	3,44	3,44	4	1	115
Dallas	88725	3,44	3,44	4	1	115
Dallas	89418	3,44	3,44	4	1	115
Dallas	92054	3,44	3,44	4	1	115
ICBM	00448	4	4	5,50	1	128
ICBM	00623	4	4	5,50	1	128
ICBM	02382	4	4	4	1	128
ICBM	02503	4	4	5,50	1	128
ICBM	05208	4	4	5,50	1	128
ICBM	07286	4	4	4	1	128
ICBM	08255	4	4	4	1	128
ICBM	08806	4	4	4	1	128
ICBM	09539	4	4	5,50	1	128
ICBM	10582	4	4	5,50	1	128
ICBM	13384	4	4	4	1	128
ICBM	13478	4	4	4	1	128
ICBM	13789	4	4	5,50	1	128
ICBM	16607	4	4	4	1	128
ICBM	19395	4	4	4	1	128
ICBM	20718	4	4	4	1	128
ICBM	22674	4	4	5,50	1	128
ICBM	26183	4	4	5,50	1	128
ICBM	26796	4	4	4	1	128
ICBM	28422	4	4	4	1	128
ICBM	28795	4	4	4	1	128
ICBM	28808	4	4	5,50	1	128
ICBM	29353	4	4	5,50	1	128
ICBM	30003	4	4	4	1	128
ICBM	30623	4	4	5,50	1	128
ICBM	32549	4	4	4	1	128
ICBM	33677	4	4	4	1	128

ICBM	34252	4	4	5,50	1	128
ICBM	35262	4	4	5,50	1	128
ICBM	35370	4	4	4	1	128
ICBM	37140	4	4	4	1	128
ICBM	40217	4	4	4	1	128
ICBM	40482	4	4	4	1	128
ICBM	41546	4	4	5,50	1	128
ICBM	41764	4	4	4	1	128
ICBM	44077	4	4	4	1	128
ICBM	44395	4	4	5,50	1	128
ICBM	47658	4	4	4	1	128
ICBM	47753	4	4	4	1	128
ICBM	48210	4	4	4	1	128
ICBM	48830	4	4	5,50	1	128
ICBM	49215	4	4	4	1	128
ICBM	51677	4	4	5,50	1	128
ICBM	53282	4	4	5,50	1	128
ICBM	53801	4	4	4	1	128
ICBM	54887	4	4	5,50	1	128
ICBM	55114	4	4	4	1	128
ICBM	55656	4	4	4	1	128
ICBM	57738	4	4	5,50	1	128
ICBM	59589	4	4	4	1	128
ICBM	59739	4	4	4	1	128
ICBM	59914	4	4	4	1	128
ICBM	62937	4	4	5,50	1	128
ICBM	63280	4	4	5,50	1	128
ICBM	65921	4	4	4	1	128
ICBM	66085	4	4	4	1	128
ICBM	66794	4	4	4	1	128
ICBM	68850	4	4	5,50	1	128
ICBM	70595	4	4	4	1	128
ICBM	71932	4	4	5,50	1	128
ICBM	72135	4	4	4	1	128
ICBM	73490	4	4	5,50	1	128

ICBM	76325	4	4	5,50	1	128
ICBM	76678	4	4	5,50	1	128
ICBM	77431	4	4	4	1	128
ICBM	78297	4	4	4	1	128
ICBM	82221	4	4	5,50	1	128
ICBM	82228	4	4	5,50	1	128
ICBM	82754	4	4	5,50	1	128
ICBM	85442	4	4	4	1	128
ICBM	86203	4	4	5,50	1	128
ICBM	86516	4	4	4	1	128
ICBM	86665	4	4	4	1	128
ICBM	87217	4	4	5,50	1	128
ICBM	89049	4	4	4	1	128
ICBM	92028	4	4	4	1	128
ICBM	93262	4	4	5,50	1	128
ICBM	93975	4	4	5,50	1	128
ICBM	94103	4	4	4	1	128
ICBM	94169	4	4	5,50	1	128
ICBM	94945	4	4	5,50	1	128
ICBM	95400	4	4	5,50	1	128
ICBM	95971	4	4	5,50	1	128
ICBM	97008	4	4	5,50	1	128
ICBM	98317	4	4	5,50	1	128
ICBM	98802	4	4	5,50	1	128
Leiden	01553	3,44	3,44	3,44	1	215
Leiden	01787	3,44	3,44	3,44	1	215
Leiden	04484	3,44	3,44	3,44	1	215
Leiden	08518	3,44	3,44	3,44	1	215
Leiden	09796	3,44	3,44	3,44	1	215
Leiden	10481	3,44	3,44	3,44	1	215
Leiden	12255	3,44	3,44	3,44	1	215
Leiden	13537	3,44	3,44	3,44	1	215
Leiden	18456	3,44	3,44	3,44	1	215
Leiden	19281	3,44	3,44	3,44	1	215
Leiden	28473	3,44	3,44	3,44	1	215

Leiden	30943	3,44	3,44	3,44	1	215
Leiden	36743	3,44	3,44	3,44	1	215
Leiden	38454	3,44	3,44	3,44	1	215
Leiden	39335	3,44	3,44	3,44	1	215
Leiden	40907	3,44	3,44	3,44	1	215
Leiden	52853	3,44	3,44	3,44	1	215
Leiden	52922	3,44	3,44	3,44	1	215
Leiden	56299	3,44	3,44	3,44	1	215
Leiden	57187	3,44	3,44	3,44	1	215
Leiden	58194	3,44	3,44	3,44	1	215
Leiden	99856	3,44	3,44	3,44	1	215

Dataset 2: high-quality task-based data, 80 sessions, 4 sessions per subject, 8.000 datapoints; for more information, see Máté *et al.*, 2016, https://dx.doi.org/10.1007/s00221-015-4473-8.

Site	ID	Resolution in X [mm]	Resolution in Y [mm]	Resolution in Z [mm]	Sessions [n]	Datapoints [n]
Tübingen	opt_002	3	3	3	4	100
Tübingen	opt_003	3	3	3	4	100
Tübingen	opt_005	3	3	3	4	100
Tübingen	opt_006	3	3	3	4	100
Tübingen	opt_007	3	3	3	4	100
Tübingen	opt_009	3	3	3	4	100
Tübingen	opt_010	3	3	3	4	100
Tübingen	opt_011	3	3	3	4	100
Tübingen	opt_012	3	3	3	4	100
Tübingen	opt_013	3	3	3	4	100
Tübingen	opt_015	3	3	3	4	100
Tübingen	opt_017	3	3	3	4	100
Tübingen	opt_018	3	3	3	4	100
Tübingen	opt_019	3	3	3	4	100
Tübingen	opt_020	3	3	3	4	100
Tübingen	opt_021	3	3	3	4	100
Tübingen	opt_022	3	3	3	4	100
Tübingen	opt_024	3	3	3	4	100
Tübingen	opt_025	3	3	3	4	100
Tübingen	opt_027	3	3	3	4	100

<u>Dataset 3</u>: low-quality task-based data, 100 sessions, up to 4 sessions per subject, 10.000 datapoints; for more information, see Wilke *et al.*, 2011 (https://doi.org/10.1002/hbm.21156) and Wilke *et al.*, 2018 (https://doi.org/10.1002/acn3.658)

Site	ID	Resolution in X [mm]	Resolution in Y [mm]	Resolution in Z [mm]	Sessions [n]	Datapoints [n]
Tübingen	paMRic_0001	3	3	3	2	100
Tübingen	paMRic_0003	3	3	3	3	100
Tübingen	paMRic_0004	3	3	3	1	100
Tübingen	paMRic_0005	3	3	3	2	100
Tübingen	paMRic_0006	3	3	3	3	100
Tübingen	paMRic_0007	3	3	3	4	100
Tübingen	paMRic_0008	3	3	3	4	100
Tübingen	paMRic_0009	3	3	3	1	100
Tübingen	paMRic_0010	3	3	3	1	100
Tübingen	paMRic_0011	3	3	3	2	100
Tübingen	paMRic_0012	3	3	3	3	100
Tübingen	paMRic_0013	3	3	3	2	100
Tübingen	paMRic_0014	3	3	3	1	100
Tübingen	paMRic_0015	3	3	3	1	100
Tübingen	paMRic_0016	3	3	3	3	100
Tübingen	paMRic_0017	3	3	3	3	100
Tübingen	paMRic_0019	3	3	3	2	100
Tübingen	paMRic_0020	3	3	3	1	100
Tübingen	paMRic_0021	3	3	3	3	100
Tübingen	paMRic_0022	3	3	3	1	100
Tübingen	paMRic_0023	3	3	3	3	100
Tübingen	paMRic_0024	3	3	3	3	100
Tübingen	paMRic_0025	3	3	3	4	100
Tübingen	paMRic_0026	3	3	3	3	100
Tübingen	paMRic_0027	3	3	3	3	100
Tübingen	paMRic_0028	3	3	3	3	100
Tübingen	paMRic_0029	3	3	3	1	100
Tübingen	paMRic_0030	3	3	3	3	100
Tübingen	paMRic_0031	3	3	3	2	100
Tübingen	paMRic_0033	3	3	3	3	100
Tübingen	paMRic_0034	3	3	3	2	100

Tübingen	paMRic_0035	3	3	3	3	100
Tübingen	paMRic_0036	3	3	3	2	100
Tübingen	paMRic_0037	3	3	3	3	100
Tübingen	paMRic_0038	3	3	3	1	100
Tübingen	paMRic_0039	3	3	3	3	100
Tübingen	paMRic_0041	3	3	3	2	100
Tübingen	paMRic_0042	3	3	3	3	100
Tübingen	paMRic_0043	3	3	3	1	100
Tübingen	paMRic_0044	3	3	3	2	100
Tübingen	paMRic_0047	3	3	3	3	100
Tübingen	paMRic_0048	3	3	3	2	100
Tübingen	paMRic_0054	3	3	3	2	100
